# Variability and magnitude of brain glutamate levels in schizophrenia: a meta and mega-analysis

**DOI:** 10.1038/s41380-023-01991-7

**Published:** 2023-02-17

**Authors:** Kate Merritt, Robert A. McCutcheon, André Aleman, Sarah Ashley, Katherine Beck, Wolfgang Block, Oswald J. N. Bloemen, Faith Borgan, Christiana Boules, Juan R. Bustillo, Aristides A. Capizzano, Jennifer M. Coughlin, Anthony David, Camilo de la Fuente-Sandoval, Arsime Demjaha, Kara Dempster, Kim Q. Do, Fei Du, Peter Falkai, Beata Galińska-Skok, Jürgen Gallinat, Charles Gasparovic, Cedric E. Ginestet, Naoki Goto, Ariel Graff-Guerrero, Beng-Choon Ho, Oliver Howes, Sameer Jauhar, Peter Jeon, Tadafumi Kato, Charles A. Kaufmann, Lawrence S. Kegeles, Matcheri S. Keshavan, Sang-Young Kim, Bridget King, Hiroshi Kunugi, J. Lauriello, Pablo León-Ortiz, Edith Liemburg, Meghan E. Mcilwain, Gemma Modinos, Elias Mouchlianitis, Jun Nakamura, Igor Nenadic, Dost Öngür, Miho Ota, Lena Palaniyappan, Christos Pantelis, Tulsi Patel, Eric Plitman, Sotirios Posporelis, Scot E. Purdon, Jürgen R. Reichenbach, Perry F. Renshaw, Francisco Reyes-Madrigal, Bruce R. Russell, Akira Sawa, Martin Schaefer, Dikoma C. Shungu, Stefan Smesny, Jeffrey A. Stanley, James Stone, Agata Szulc, Reggie Taylor, Katharine N. Thakkar, Jean Théberge, Philip G. Tibbo, Thérèse van Amelsvoort, Jerzy Walecki, Peter C. Williamson, Stephen J. Wood, Lijing Xin, Hidenori Yamasue, Philip McGuire, Alice Egerton, the 1H-MRS in Schizophrenia Investigators, Camilo de la Fuente-Sandoval, Thérèse van Amelsvoort, Philip K. McGuire

**Affiliations:** 1 Division of Psychiatry, UCL, Institute of Mental Health, London, UK; 2 Dept of Psychiatry, Warneford Hospital, University of Oxford, Oxford, UK; 3 Center for Brain Disorder and Cognitive Science, Shenzhen University, Shenzhen, China; 4 University Medical Center Groningen, University of Groningen, Groningen, the Netherlands; 5 Psychosis Studies Department, Institute of Psychiatry, Psychology and Neuroscience, King’s College London, London, UK; 6 Department of Diagnostic and Interventional Radiology, University Hospital Bonn, Bonn, Germany; 7 Department of Psychiatry and Neuropsychology, Maastricht University, Maastricht, the Netherlands; 8 Department of Psychiatry and Behavioral Sciences, Center for Psychiatric Research, University of New Mexico School of Medicine, Albuquerque, NM USA; 9 Department of Radiology, Division of Neuroradiology, University of Michigan, 1500 E Medical Center Dr, Ann Arbor, MI 48109 USA; 10 Department of Psychiatry and Behavioral Sciences, Johns Hopkins University School of Medicine, Baltimore, MD USA; 11 Laboratory of Experimental Psychiatry, Instituto Nacional de Neurología y Neurocirugía, Mexico City, Mexico; 12 Neuropsychiatry Department, Instituto Nacional de Neurología y Neurocirugía, Mexico City, Mexico; 13 Department of Psychiatry, Dalhousie University, Halifax, NS Canada; 14 Center for Psychiatric Neuroscience (CNP), Department of Psychiatry, Lausanne University Hospital-CHUV, Prilly-Lausanne, Switzerland; 15 Psychotic Disorders Division, McLean Hospital, Harvard Medical School, Belmont, MA USA; 16 Department of Psychiatry, University Hospital, LMU Munich, Nussbaumstrasse 7, 80336 Munich, Germany; 17 Department of Psychiatry, Medical University of Bialystok, Bialystok, Poland; 18 Department of Psychiatry and Psychotherapy, University Medical Center Hamburg-Eppendorf (UKE), Hamburg, Germany; 19 Mind Research Network, Albuquerque, NM USA; 20 Department of Biostatistics and Health Informatics (S2.06), Institute of Psychiatry, Psychology and Neuroscience King’s College London, London, UK; 21 Department of Psychiatry, Kokura Gamo Hospital, Kitakyushu, Fukuoka 8020978 Japan; 22 Multimodal Neuroimaging Schizophrenia Group, Research Imaging Centre, Geriatric Mental Health Program at Centre for Addiction and Mental Health, and Department of Psychiatry, University of Toronto, Toronto, ON Canada; 23 Department of Psychiatry, University of Iowa Carver College of Medicine, Iowa City, IA USA; 24 Department of Medical Biophysics, University of Western Ontario, London, ON Canada; 25 Department of Psychiatry and Behavioral Science, Juntendo University Graduate School of Medicine, Tokyo, Japan; 26 Department of Psychiatry, Columbia University, New York State Psychiatric Institute (NYSPI), New York, NY USA; 27 Columbia University, Department of Psychiatry, New York State Psychiatric Institute (NYSPI), New York, NY USA; 28 Harvard Medical School, 75 Fenwood Rd., Boston, MA 02115 USA; 29 Philips Healthcare, Seoul, 0463 Republic of Korea; 30 National Center of Neurology and Psychiatry, Kodaira, Tokyo 187-0031 Japan; 31 Jefferson Health-Sidney Kimmel Medical College, Philadelphia, PA USA; 32 Rob Giel Research Center, Department of Psychiatry, University Medical Center Groningen, Groningen, the Netherlands; 33 School of Pharmacy, University of Auckland, 85 Park Road, Grafton, Auckland, 1023 New Zealand; 34 Department of Neuroimaging, Centre for Neuroimaging Sciences, Institute of Psychiatry, Psychology & Neuroscience, De Crespigny Park, London, SE5 8AF UK; 35 Department of Psychiatry, University of Occupational and Environmental Health, Kitakyushu, Fukuoka Japan; 36 Douglas Mental Health University Institute, Department of Psychiatry, McGill University, Montreal, QC Canada; 37 Melbourne Neuropsychiatry Centre, The University of Melbourne and Melbourne Health, Carlton, VIC Australia; 38 The Florey Institute of Neuroscience and Mental Health, Parkville, VIC Australia; 39 Cerebral Imaging Centre, Douglas Mental Health University Institute, Montreal, QC Canada; 40 Department of Psychiatry, McGill University, Montreal, QC Canada; 41 South London and Maudsley, Bethlem Royal Hospital, Monks Orchard Road, Beckenham, BR3 3BX UK; 42 Neuropsychology Department, Alberta Hospital Edmonton, Edmonton, AB Canada; 43 Department of Psychiatry, University of Alberta, Edmonton, AB Canada; 44 Medical Physics Group, Institute for Diagnostic and Interventional Radiology (IDIR), Jena University Hospital, Jena, Germany; 45 Department of Psychiatry, University of Utah, Salt Lake City, UT USA; 46 School of Pharmacy, University of Otago, Dunedin, New Zealand; 47 Departments of Psychiatry, Neuroscience, Mental Health, Biomedical Engineering, and Genetic Medicine, Johns Hopkins University, Baltimore, MD USA; 48 Department of Psychiatry, Psychotherapy, Psychosomatics and Addiction Medicine, Kliniken Essen-Mitte, Essen, Germany; 49 Department of Psychiatry and Psychotherapy, Charité-Universitätsmedizin Berlin, Campus Charité Mitte, Berlin, Germany; 50 Department of Radiology, Weill Cornell Medical College, New York City, NY USA; 51 Department of Psychiatry and Psychotherapy, Jena University Hospital, Jena, Germany; 52 Brain Imaging Research Division, Department of Psychiatry and Behavioral Neurosciences, Wayne State University School of Medicine, Detroit, MI USA; 53 Brighton and Sussex Medical School, University of Sussex, Brighton, UK; 54 Department of Psychiatry, Medical University of Warsaw, Warsaw, Poland; 55 Lawson Health Research Institute, London, ON Canada; 56 Department of Psychology, Michigan State University, East Lansing, MI USA; 57 Division of Psychiatry and Behavioral Medicine, Michigan State University, East Lansing, MI USA; 58 Department of Psychiatry, Western University, London, ON Canada; 59 Postgraduate Medical School, Warsaw, Poland; 60 Orygen, Melbourne, VIC Australia; 61 Institute for Mental Health, University of Birmingham, Edgbaston, UK; 62 Centre for Youth Mental Health, University of Melbourne, Melbourne, VIC Australia; 63 Animal Imaging and Technology Core (AIT), Center for Biomedical Imaging (CIBM), Ecole Polytechnique Fédérale de Lausanne, Lausanne, Switzerland; 64 Department of Psychiatry, Hamamatsu University School of Medicine, Hamamatsu, Japan

**Keywords:** Neuroscience, Schizophrenia

## Abstract

Glutamatergic dysfunction is implicated in schizophrenia pathoaetiology, but this may vary in extent between patients. It is unclear whether inter-individual variability in glutamate is greater in schizophrenia than the general population. We conducted meta-analyses to assess (1) variability of glutamate measures in patients relative to controls (log coefficient of variation ratio: CVR); (2) standardised mean differences (SMD) using Hedges g; (3) modal distribution of individual-level glutamate data (Hartigan’s unimodality dip test). MEDLINE and EMBASE databases were searched from inception to September 2022 for proton magnetic resonance spectroscopy (1H-MRS) studies reporting glutamate, glutamine or Glx in schizophrenia. 123 studies reporting on 8256 patients and 7532 controls were included. Compared with controls, patients demonstrated greater variability in glutamatergic metabolites in the medial frontal cortex (MFC, glutamate: CVR = 0.15, *p* < 0.001; glutamine: CVR = 0.15, *p* = 0.003; Glx: CVR = 0.11, *p* = 0.002), dorsolateral prefrontal cortex (glutamine: CVR = 0.14, *p* = 0.05; Glx: CVR = 0.25, *p* < 0.001) and thalamus (glutamate: CVR = 0.16, *p* = 0.008; Glx: CVR = 0.19, *p* = 0.008). Studies in younger, more symptomatic patients were associated with greater variability in the basal ganglia (BG glutamate with age: *z* = −0.03, *p* = 0.003, symptoms: *z* = 0.007, *p* = 0.02) and temporal lobe (glutamate with age: *z* = −0.03, *p* = 0.02), while studies with older, more symptomatic patients associated with greater variability in MFC (glutamate with age: *z* = 0.01, *p* = 0.02, glutamine with symptoms: *z* = 0.01, *p* = 0.02). For individual patient data, most studies showed a unimodal distribution of glutamatergic metabolites. Meta-analysis of mean differences found lower MFC glutamate (*g* = −0.15, *p* = 0.03), higher thalamic glutamine (*g* = 0.53, *p* < 0.001) and higher BG Glx in patients relative to controls (*g* = 0.28, *p* < 0.001). Proportion of males was negatively associated with MFC glutamate (*z* = −0.02, *p* < 0.001) and frontal white matter Glx (*z* = −0.03, *p* = 0.02) in patients relative to controls. Patient PANSS total score was positively associated with glutamate SMD in BG (*z* = 0.01, *p* = 0.01) and temporal lobe (*z* = 0.05, *p* = 0.008). Further research into the mechanisms underlying greater glutamatergic metabolite variability in schizophrenia and their clinical consequences may inform the identification of patient subgroups for future treatment strategies.

## Introduction

Several lines of evidence implicate glutamatergic dysfunction in the pathoaetiology of schizophrenia [1]. It is not clear, however, whether the degree of glutamatergic dysfunction is similar across individuals with schizophrenia or whether there is significant interindividual variability over and above the variability observed in the general population. Meta-analyses of 1H-MRS studies report higher glutamate and combined glutamate and glutamine (Glx) in the basal ganglia [2, 3], higher glutamine in the thalamus [2, 3] and lower glutamate in the medial frontal cortex (MFC) [3–6] in schizophrenia in comparison to controls. Two meta-analyses do not report lower glutamate levels in the medial frontal cortex [2, 3] but find lower levels in non treatment-resistant patients [3]. Glutamate levels in schizophrenia show heritability [7], are associated with glutamatergic genetic risk [8] and may also be altered by environmental factors [9]. There is some evidence that glutamate levels are positively associated with the severity of symptoms of schizophrenia [10–13] and may be reduced by treatment with antipsychotic medication [12, 14]. It is unknown, however, whether these associations are similar across all patients or if they vary between individuals, for example due to differences in underlying neurobiology or illness stage. This could be important if heterogeneity in glutamate measures is related to treatment outcomes in schizophrenia. For example, there is already some evidence that elevated glutamate levels may be most apparent in patients whose symptoms do not respond well to antipsychotic medication [15–24], and that glutamate-acting compounds could have selective efficacy in some patient subgroups [25]. If greater variability is present, it is of interest as to whether this manifests in the form of a spectrum (following a unimodal distribution), or whether there is evidence of a bimodal distribution, consistent with discrete subtypes of schizophrenia with different brain glutamate levels [15].

The hypothesis of greater glutamate variability in patients can be formally tested by conducting a meta-analysis of variability, as previously employed to examine the variability of glutathione [26], antipsychotic treatment response [27] and brain structure in schizophrenia [28]. One meta-analysis examined the variability of glutamate in schizophrenia compared to controls, but this was limited to glutamate in the dorsolateral prefrontal cortex (DLPFC) [29]. In addition, the hypothesis of discrete subgroups can be tested by examining the distribution of individual patient data [30].

In the current meta-analysis, we hypothesised that patients with schizophrenia would exhibit greater variability of brain glutamate, glutamine and Glx levels than controls. We complemented this with analysis of the distribution of individual-level data, as a bimodal distribution of glutamatergic metabolites in patients would support the existence of discrete glutamate subgroups. Additionally, we present an updated meta-analysis of case-control differences in glutamate metabolites to include recently published data that has not been previously summarised. In accordance with recent meta-analyses, we hypothesised that glutamine levels in thalamus and glutamate and Glx levels in the basal ganglia will be higher in schizophrenia patients compared to controls, and that MFC glutamate will be lower. Sensitivity analyses examined whether variability or standardised mean differences (SMD) in glutamatergic metabolites were associated with antipsychotic medication exposure, and meta-regressions tested for potential effects of age, sex, symptom severity and antipsychotic medication dose. On the basis of results from a recent mega-analysis [12], we hypothesised that age will not be associated with SMD, whereas medication exposure will be associated with negative effect sizes and symptom severity with positive effect size differences between patients and controls. Meta-regressions of the same variables were performed to investigate potential sources of patient-control differences in variability.

## Methods

### Search strategy and study selection

We followed PRISMA guidelines and registered the study on PROSPERO (CRD42021251798). MEDLINE and EMBASE databases were searched to identify articles published from inception to September 23, 2022, using the search terms: (1) MRS or magnetic resonance spectroscopy AND (2) schizophrenia or psychosis or UHR or ARMS or schizoaffective or “Psychosis risk” OR “at risk mental state” or “psychotic experience” or “psychosis spectrum” OR (“genetic risk” and (psychosis or schizophrenia)) OR (“high risk” and (psychosis or schizophrenia)). Screening and selection of studies was performed independently by three authors (KM, KB, CB). 1H-MRS studies reporting glutamate, glutamine, or Glx values for a schizophrenia patient group in comparison with a healthy volunteer group were included in the analysis. Studies in clinical high risk or genetic high risk cohorts have been summarised elsewhere [31] and were excluded from the current analysis on individuals meeting diagnostic criteria. In the case of longitudinal studies, only the values for the first time point were included. If the same sample or partially overlapping samples were included in more than one report, data from the study with the largest sample were included. Where the mean or standard deviations for glutamate measures were unavailable in the published manuscript the authors were contacted and values requested. We also requested individual patient level datasets from authors as part of a previous mega-analysis (for further details see 12).

### Data extraction and processing

Mean and standard deviation (SD) values of glutamate, glutamine, or Glx concentrations were extracted (K.M.) and verified independently (S.A. and B.K.), and categorized into the following brain regions of interest: (1) medial frontal cortex (MFC), including voxels in the medial prefrontal cortex and in the anterior cingulate cortex since these voxels often spatially overlap; (2) dorsolateral prefrontal cortex (DLPFC); (3) frontal white matter; (4) thalamus; (5) temporal lobe (including superior temporal gyrus and hippocampus); (6) basal ganglia (including caudate, putamen, globus pallidus and substantia nigra). When more than one clinical group was reported in a single study, the values were treated as independent data sets and the number of controls was adjusted by dividing by the number of clinical groups. We also extracted participant age, sex, Positive and Negative Syndrome Scale (PANSS) scores and antipsychotic dose in chlorpromazine equivalents (CPZ), and the SD values for these variables (data available in Supplementary eTable 1. Data and R code also available on github [32]). The quality of included studies was rated using the Newcastle-Ottawa Scale [33] (Supplementary eAppendix 1). Metabolite measures using J-edited and echo-planar spectroscopic imaging (EPSI) acquisition sequences were scaled by 1000 [34–36] or 100,000 to obtain comparable values [37].

### Meta-analysis outcome measures

The relative variability between patients and controls can be quantified using the log variability ratio (VR), taking into account the standard deviation of mean glutamate values. In many natural systems mean scales with variability, and if this is not accounted for, variability differences can be influenced by mean differences. We therefore used the log coefficient of variation ratio (CVR) for our primary analyses, which adjusts the VR for mean differences between groups:

$$\ln {{{{{{{\mathrm{CVR}}}}}}}} = {{{{{{{\mathrm{ln}}}}}}}}\left( {\frac{{\hat \sigma _p/\bar x_p}}{{\hat \sigma _c/\bar x_c}}} \right) = {{{{{{{\mathrm{ln}}}}}}}}\left( {\frac{{S_p/\bar x_p}}{{S_c/\bar x_c}}} \right) + \frac{1}{{2\left( {n_p - 1} \right)}} - \frac{1}{{2(n_c - 1)}}$$

Where $\hat \sigma _p$ and $\hat \sigma _c$ are the unbiased estimates of the population standard deviation for the patient and control groups respectively, $\bar x_p$ and $\bar x_c$ are the mean values, *S*_*p*_ and *S*_*c*_ are the reported SDs, while *n*_*p*_ and *n*_*c*_ are the sample sizes.

A CVR above 0 indicates greater variability in patients, and below 0 indicates greater variability in controls. While CVR is used for our primary analysis, VR is presented in the supplementary results.

The standardised mean differences (Hedges’ g) of glutamatergic metabolites between patients and controls were calculated using a random effects model. A Hedges’ g value of 0 indicates no difference between patients and controls, negative values indicate lower glutamatergic metabolite levels in patients than controls, and positive values denote higher metabolite levels in patients than controls. I^2^ values were calculated to quantify between-study inconsistency. Benjamini-Hochberg false discovery rate (FDR) was used to correct for the number of regions (Q false discovery rate of 10%).

Sensitivity analyses examined CVR and Hedges’ g effect sizes in antipsychotic-naïve and medicated patients separately, which were then compared in a Wald type test to assess significance. Meta-regressions assessed the impact of the combined mean age of sample (patients and controls), proportion of males in combined sample, PANSS total score and CPZ on CVR and Hedges’ g effect sizes. Significant meta-regressions were followed up with meta-regressions to determine if the SD of a demographic/clinical variable was associated with CVR. Significant CVR results were followed up with meta-regressions to determine if CVR was associated with 1H-MRS data quality, namely (1) signal to noise ratio (SNR) (patient mean divided by control mean) and (2) field homogeneity (linewidth as full width half maximum values: FWHM) (patient mean divided by control mean). Analyses were carried out in R (version 4.1.1), using the “metafor” [38] and “weights” packages, while plots were generated using “ggplot2” [39].

### Data distribution

The modal distribution of data was investigated in individual-level patient data from 33 studies contributed by the 1H-MRS in Schizophrenia Investigators consortium (for further details see 12). For each study, data was normalized (mean-scaled) and analysed using Hartigan’s Dip Test of Unimodality [30] (R package “dip.test”).

## Results

### Study selection

The search identified 2527 articles, 123 of which met criteria for the meta-analysis (Supplementary eFig. 1 and eResults 1 for references), including data on 8256 patients with schizophrenia and 7532 controls. The average age was 31 years and males constituted 66% of participants (Supplementary eTable 1 for raw data). Studies examined first episode psychosis patients (57 studies), patients with established schizophrenia (72 studies) and antipsychotic-naïve patients (33 studies).

### Variability analyses

#### Meta-analysis of CVR

There was a positive relationship between mean glutamatergic metabolite level and standard deviation (weighted rp = 0.70, *p* < 0.001, Supplementary eFig. 2), indicating mean-scaling of variability and the appropriateness of CVR as the primary variability outcome measure.

**Fig. 1:**
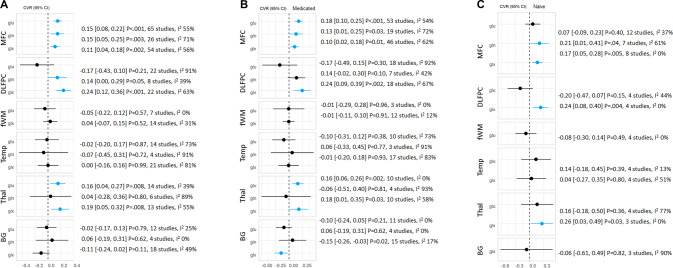
Forest plot showing the summary effect sizes for the coefficient of variation ratio (CVR) of glutamate measures. **A** CVR in schizophrenia patients compared to healthy volunteers (HV). **B** CVR in patients treated with antipsychotic medication compared to HV. **C** CVR in antipsychotic-naïve patients compared to HV. Significant results are shown in blue. Variability was significantly higher in patients relative to HV in the medial frontal cortex (MFC; all glutamatergic metabolites), dorsolateral prefrontal cortex (DLPFC: Glutamine and Glx) and Thalamus (Thal; Glutamate and Glx-). There were no significant differences in glutamatergic metabolite variability in the frontal white matter (fWM), temporal lobe (Temp) and basal ganglia (BG) in patients compared with HV. Reduced Glx variability in the basal ganglia (BG) was found in medicated patients relative to HV. CVR, 95% confidence intervals, *P* value and I^2^ presented.

In the MFC, variability of glutamate, glutamine and Glx were significantly increased in patients compared with controls (CVR = 0.15, 95% CI 0.08–0.22, *p* < 0.001, 65 studies; CVR = 0.15, 95% CI 0.05–0.25, *p* = 0.003, 26 studies; CVR = 0.11, 95% CI 0.04–0.18, *p* = 0.002, 54 studies respectively; Fig. 1). In the DLPFC, variability of glutamine and Glx were significantly increased in patients compared with controls (CVR = 0.14, 95% CI 0.00–0.29, *p* = 0.05, 8 studies and CVR = 0.24, 95% CI 0.12–0.36, *p* < 0.001, 22 studies), but the variability of glutamate did not differ (Fig. 1). In the thalamus, variability of glutamate and Glx were significantly increased in patients compared with controls (CVR = 0.16, 95% CI 0.04–0.27, *p* = 0.008, 14 studies and CVR = 0.19, 95% CI 0.05–0.32, *p* = 0.008, 13 studies), but the variability of glutamine did not differ (Fig. 1). For the frontal white matter, basal ganglia and temporal lobe, the variability of glutamatergic metabolite measures did not differ between patients and controls (Fig. 1). All findings survived FDR correction.

Sensitivity analyses examined antipsychotic-naïve and medicated patients separately, and produced the same pattern of results for the MFC and DLPFC (Fig. 1), although the variability of glutamate did not differ between antipsychotic-naïve patients and healthy volunteers in the MFC. In the thalamus, when analysis was restricted to antipsychotic-naïve patients the variability of glutamate no longer differed between groups (Fig. 1). In the basal ganglia, Glx variability was reduced in medicated patients compared with controls (CVR = −0.15, 95% CI −0.26 to −0.03, *p* = 0.02, 15 studies), but this reduction was not apparent in antipsychotic-naïve patients (3 studies) or across the whole sample. Significant differences in CVR were not found between antipsychotic-naïve and medicated patients in the MFC (glutamate), thalamus (glutamate) and basal ganglia (Glx).

Log VR results were largely the same as CVR and are reported in the Supplement (eFig. 3).

#### Meta-regressions of CVR

**Fig. 2:**
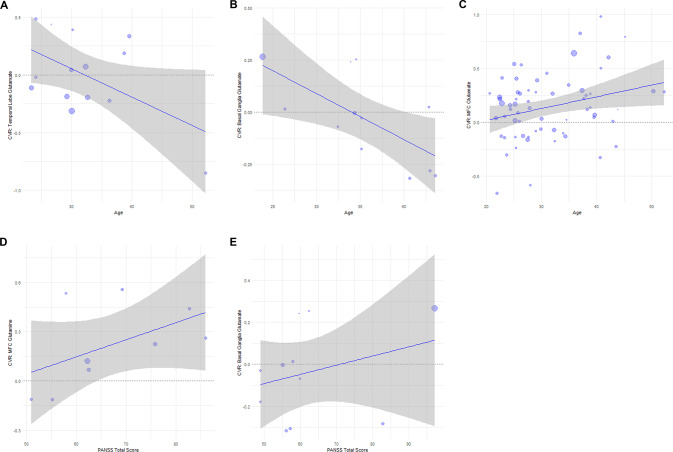
Meta-regressions of the coefficient of variation ratio (CVR) and age or PANSS total score. **A** In the temporal lobe and **B** basal ganglia, higher glutamate variability in patients relative to HV was associated with studies in younger participants. **C** In the medial frontal cortex (MFC), higher glutamate variability in patients relative to HV was associated with studies in older participants. For glutamine in the **D** MFC and **E** glutamate in the basal ganglia, higher variability in patients relative to healthy volunteers (HV) was associated with studies in more symptomatic patients (according to PANSS Total Scores). Bubble size represents total sample of patients and HV.

PANSS total score was positively associated with glutamine variability (CVR) in the MFC (*z* = 0.01, *p* = 0.02, 9 studies, Fig. 2) and glutamate variability in the basal ganglia (*z* = 0.007, *p* = 0.02, 11 studies), indicating that studies examining patients with a greater symptom severity showed greater glutamatergic variability in patients compared to controls. Age (of patients and controls combined) was negatively associated with glutamate variability in the temporal lobe (*z* = −0.03, *p* = 0.02, 14 studies) and basal ganglia (*z* = −0.03, *p* = 0.003, 12 studies), indicating that studies examining younger participants showed greater variability in the patient group relative to controls. Age (of patients and controls combined) was positively associated with glutamate variability in the MFC (*z* = 0.01, *p* = 0.02, 65 studies). CPZ and proportion of males were not significantly related with CVR.

The SD of PANSS total score for each study was not associated with glutamine variability in the MFC or glutamate variability in the basal ganglia, indicating that the association between PANSS total score and CVR is not explained by increased variability in PANSS total score. Similarly, there was no association between age SD and glutamate CVR in the basal ganglia. In the temporal lobe, greater age SD was associated with reduced CVR in glutamate (*z* = −0.05, *p* = 0.05, 14 studies), suggesting that greater glutamate CVR in younger patients is unlikely to be explained by greater variability in age in younger cohorts. 1H-MRS data quality measures (SNR and FWHM) were not associated with CVR in the MFC, DLPFC or thalamus.

#### Distribution of individual patient glutamate and Glx data

27 studies contributed individual participant-level glutamatergic metabolite data in the MFC. 18 studies were in patients previously or currently treated with antipsychotics, 6 studies were in antipsychotic-naïve or minimally medicated cohorts, and 3 studies included both antipsychotic-naïve and medicated patients. Hartigan’s dip test found unimodality in the distribution of mean-centred Cr-scaled glutamate (*D* = 0.01, *p* = 0.99) and Glx in patients (*D* = 0.01, *p* = 0.99), and for CSF-corrected glutamate (*D* = 0.02, *p* = 0.37) and Glx in patients (*D* = 0.01, *p* = 0.68). Hartigan’s dip test for individual studies found unimodality in 26 studies [15, 16, 21, 22, 40–60] and bimodality in 1 study [61].

For the temporal lobe, 6 studies contributed individual participant-level glutamatergic metabolite data. 4 studies were in patients previously or currently treated with antipsychotics and 2 studies were in antipsychotic-naïve or minimally medicated cohorts. Hartigan’s dip test found unimodality in the distribution of mean-centred CSF-corrected Glx (*D* = 0.04, *p* = 0.581, 3 studies) and Cr-scaled Glx in patients (*D* = 0.03, *p* = 0.68, 6 studies). Hartigan’s dip test for individual studies found unimodality in all 6 studies [51, 53, 62–65].

### Standardised mean differences analyses

#### Meta-analysis of hedges’ g effect sizes

**Fig. 3:**
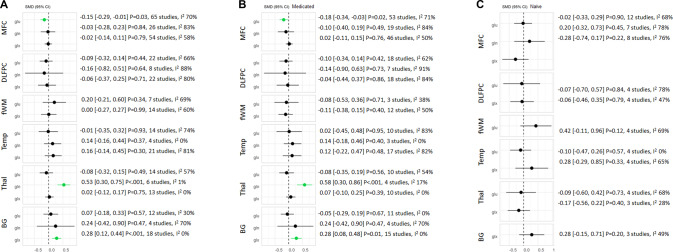
Forest plot showing summary Hedges’ g effect sizes for standardised mean differences (SMD) of glutamate measures. **A** SMD in schizophrenia patients compared to healthy volunteers (HV). **B** SMD in patients treated with antipsychotic medication compared to HV. **C** SMD in antipsychotic-naïve patients compared to HV. Significant results are shown in green. Glutamate levels in the medial frontal cortex (MFC) were significantly lower in patients relative to HV, whereas glutamine levels in the thalamus (Thal) and Glx levels in the basal ganglia (BG) were significantly higher in patients relative to HV. There were no significant differences in glutamatergic metabolite levels in the dorsolateral prefrontal cortex (DLPFC), frontal white matter (fWM) or temporal lobe (Temp) between patients and HV. Significant differences in glutamatergic metabolites were no longer present when antipsychotic-naïve patients were assessed. Hedges’ g, 95% confidence intervals, *P* value and I^2^ presented.

In the MFC, glutamate levels were significantly lower in patients compared with controls (*g* = −0.15, 95% CI −0.29 to −0.01, *p* = 0.03, 65 studies, Fig. 3), whereas glutamine and Glx levels did not differ (Fig. 3). In the thalamus, glutamine levels were significantly higher in patients compared with controls (*g* = 0.53, 95% CI 0.30–0.75, *p* < 0.001, 6 studies), and glutamate and Glx levels did not differ (Fig. 3). In the basal ganglia, Glx levels were significantly higher in patients compared with controls (*g* = 0.28, 95% CI 0.12–0.44, *p* < 0.001, 18 studies) and glutamate and glutamine did not differ (Fig. 3). For the DLPFC, frontal white matter and temporal lobe, glutamatergic metabolite levels did not differ between patients and controls (Fig. 3). All findings survived FDR correction except glutamate in the MFC.

The same pattern of results was apparent when analyses were restricted to medicated patients (Fig. 3). When analyses were restricted to antipsychotic-naïve patients there were no significant differences in glutamatergic metabolites. However, there were no significant differences in Hedges’ g effect sizes between antipsychotic-naïve and medicated patients in the MFC (glutamate) or basal ganglia (Glx) and an insufficient number of studies to assess Hedges’ g effect sizes in antipsychotic-naïve patients for glutamine in the thalamus.

#### Meta-regressions of hedges’ g effect sizes

**Fig. 4:**
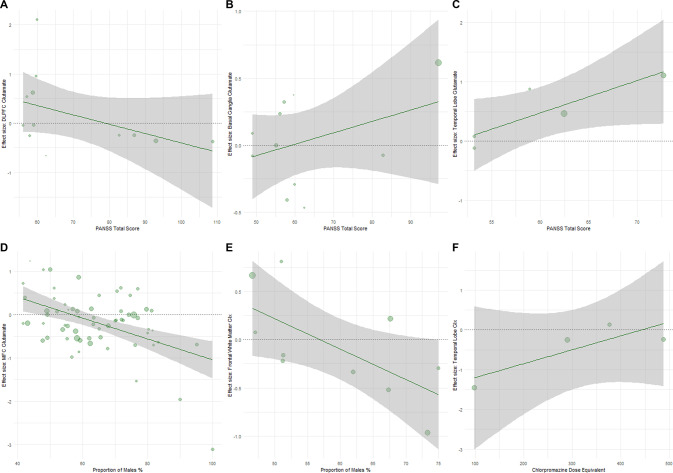
Meta-regressions of Hedges’ g effect sizes and PANSS total score or proportion of males or chlorpromazine equivalent dose (CPZ). **A** In the dorsolateral prefrontal cortex (DLPFC), lower glutamate levels in patients relative to healthy volunteers (HV) was associated with studies examining patients with greater symptom severity (according to PANSS Total Scores). In the (**B**) basal ganglia and (**C**) temporal lobe, higher glutamate levels were associated with studies examining patients with greater symptom severity (according to PANSS Total Scores). **D** In the medial frontal cortex (MFC) and (**E**) frontal white matter, lower glutamate and Glx levels in patients relative to HV was associated with studies including a higher proportion of males. **F** In the temporal lobe, lower Glx in patients relative to HV was associated with lower CPZ. Bubble size represents total sample of patients and HV.

Mean PANSS total score was positively associated with effect sizes for elevated glutamate in patients in the temporal lobe (*z* = 0.05, *p* = 0.008, 5 studies) and basal ganglia (*z* = 0.01, *p* = 0.01, 11 studies), and negatively correlated with glutamate in the DLPFC (*z* = −0.02, *p* = 0.03, 12 studies, Fig. 4). Age was negatively associated with the effect size for elevated glutamine in patients compared to controls in the basal ganglia (*z* = −0.15, *p* = 0.003, 4 studies, Supplementary eFig. 4). In frontal white matter, age was negatively associated with the effect size for elevated glutamate in patients (*z* = −0.04, *p* = 0.03, 7 studies), but positively associated with the effect size for elevated Glx (*z* = −0.02, *p* = 0.02, 12 studies). A higher proportion of males was associated with lower MFC glutamate (*z* = −0.02, *p* < 0.001, 64 studies) and frontal white matter Glx in patients compared to controls (*z* = −0.03, *p* = 0.02, 10 studies) (Fig. 4). Lower CPZ associated with effect sizes for lower Glx in patients in the temporal lobe (*z* = 0.003, *p* = 0.03). CPZ did not associate with Hedges’ g effect sizes in other brain regions.

## Discussion

The main findings of this meta-analysis are that schizophrenia is associated with increased variability in the concentrations of glutamatergic metabolites in the brain, together with regional differences in mean glutamatergic metabolite concentrations. The greatest amount of data were available for the MFC (65 studies), where glutamate levels were lower and the variability of all glutamate metabolites (glutamate, glutamine and Glx) were increased in schizophrenia compared to controls (although the finding of lower MFC glutamate in patients compared to controls did not survive FDR correction). While the emphasis has been on glutamate in the MFC in schizophrenia, our results additionally indicate glutamatergic dysregulation in other brain regions, finding increased variability in glutamine and Glx in the DLPFC, higher levels of Glx in the basal ganglia, and higher levels of glutamine alongside increased variability of glutamate and Glx in the thalamus. The increased variability in glutamatergic metabolites tended to be most apparent in studies examining patients who were more symptomatic.

Glutamate is tightly regulated in the brain through complex feedback mechanisms which may be disrupted in schizophrenia [66]. The increased variability in glutamatergic metabolites in schizophrenia indicate a spectrum of disturbances in glutamate homeostatic control between individual patients, resulting in a wide range of concentration values. Regionally, our analysis of the available data in schizophrenia indicates dysfunctional regulation (i.e. increased variability) of glutamatergic metabolite concentrations in the MFC, DLPFC and thalamus, and mean value decreases in the MFC and increases in the thalamus and basal ganglia. Increased glutamatergic variability in schizophrenia, as observed at the macro scale with 1H-MRS, could relate to individual differences in the nature or in the extent of the underlying molecular pathophysiological mechanisms. We did not find evidence for a bimodal distribution within individual participant data, as would be expected if between-subject glutamatergic differences were driven by the presence or absence of a simple, discrete variable. In contrast, the observed unimodal distributions are consistent with the view that glutamatergic pathology in schizophrenia arises secondary to a range of factors that vary amongst patients (e.g polygenic and multiple environmental vulnerability variables) [7–9]. Therefore, findings of higher glutamate levels in treatment resistant patients in comparison to treatment responders [15–24] may represent a continuum, rather than the presence of discrete subtypes.

There was evidence in some regions that the variability of glutamate or glutamine was related to symptom severity and age. In both the basal ganglia and MFC, variability was greater in studies recruiting more symptomatic patients. This could indicate that dysfunction of glutamate regulatory mechanisms is greatest in those patients with highest illness burden. These findings were not explained by increased variance in symptom scores in studies recruiting more unwell patients, or by the absence of antipsychotic medication as variance was similar in medicated compared to unmedicated cohorts. In the basal ganglia and temporal lobe, variability was higher in studies recruiting younger patients. In older patients, our results indicated that glutamate in these regions may be even more homogenous than that seen in controls, which could potentially reflect regulatory over-compensation. Alternatively, this may reflect greater clinical heterogeneity within first episode cohorts, for example in respect to diagnosis. In contrast in the MFC glutamate was more variable in studies including older patients. Potentially, this could relate to heterogeneity in clinical outcomes, as treatment response has been most strongly linked to differential glutamate levels in this region [15, 17–23]. The relationship between basal ganglia glutamate and symptom severity was largely influenced by a single study. Further studies in highly symptomatic patients are therefore needed to confirm this finding, and to determine whether treatment response contributes to increased variability in the MFC.

As increased variability was of comparable magnitudes in antipsychotic-naive and medicated patient cohorts, this suggests that variability in glutamate does not result from differential effects of antipsychotic medication on glutamate levels between individuals [14, 21]. Although a recent meta-analysis of DLPFC glutamate found higher variability in antipsychotic medicated patients and lower variability in medication-naïve patients [29]. Our analysis, including 12 more recent studies and investigating DLPFC glutamate and Glx separately, did not find any effect of medication status in this region. In fact, in the basal ganglia, it appeared that antipsychotic-treated cohorts displayed reduced variability compared to controls. Potentially this could be due to a regulatory effect of antipsychotics on basal ganglia Glx [67, 68].

The meta-analysis of standardised mean differences found lower MFC glutamate levels and higher thalamic glutamine and basal ganglia Glx in patients compared to controls, although lower MFC glutamate did not survive FDR correction. These results are consistent with recent meta-analyses [2–6], but substantially extend them by including 20 new datasets for MFC glutamate and 2 new datasets for thalamic glutamine. Stratified analyses found that lower MFC glutamate was observed across studies examining medicated patients but not across studies examining antipsychotic-naïve patients. Thus, antipsychotic medication could lower MFC glutamate levels, as indicated by longitudinal studies [14, 21] and a mega-analysis [12], although the meta-regression with CPZ dose was not significant. Our analysis also revealed relationships between the proportion of males in the study and MFC glutamate and frontal white matter Glx effect sizes, such that a higher proportion of males was associated with lower glutamate levels in patients compared to controls. In schizophrenia, males show greater elevation in peripheral d-serine (an NMDA receptor co-agonist) [69], and, in the postmortem ACC, less upregulation of glutamine synthetase [70] and lower expression of GABAergic genes encoding proteins which modulate glutamate neurotransmission [71]. The effect of sex should be further investigated on the individual level through large studies or mega-analyses. Although there was no difference in glutamate metabolite variability in the temporal lobe, higher glutamate levels were found in studies which included more highly symptomatic patients. This is also consistent with our recent mega-analysis [12]. Finally, other than in the basal ganglia and frontal white matter, meta-regressions did not find an accelerated loss of glutamatergic metabolites in patients with age for the majority of brain regions, also consistent with our recent mega-analysis [12].

A limitation of the meta-analysis is the high between-study inconsistency, as measured by I^2^, for most brain regions studied. This was highest in the temporal lobe and may relate to the difficulty of obtaining good quality 1H-MRS imaging in this region. There is a possibility that case-control differences in variability result from greater movement artifacts in patient populations [72], however CVR did not correlate with SNR or FWHM values. A recent meta-analysis emphasises the importance of using strict Cramér–Rao lower bound criteria (≤7%) and short echo times (≤20 ms) to improve 1H-MRS consistency [5]. Furthermore, the glutamine signal cannot be accurately resolved from glutamate below 3 T, although the majority of studies reporting glutamine were conducted above 3 T. As voxel placement varied between studies, broad categories of brain regions were used, limiting the regional specificity of our results. Meta-regression analyses of clinical and demographic variables are limited to the study level and are not sensitive to variation within individual studies (although meta-regressions with the SD of clinical and demographic variables were carried out). Lastly, the number of included studies is low for some brain regions, such as the thalamus, and there are a small number of studies examining antipsychotic-naïve patients in all regions except the MFC, and so these sensitivity analyses should be considered preliminary.

In summary, this meta-analysis demonstrates increased regional variability in glutamatergic metabolites in schizophrenia in addition to mean differences compared to controls. Increased inter-individual differences in glutamatergic metabolites in schizophrenia are likely to have a complex mechanistic basis. Further work is also required to determine the clinical consequences along the spectrum of glutamate dysregulation. Both glutamatergic metabolite levels [12] and interindividual variability appear to be greater in more symptomatic patients. Neurobiological heterogeneity may also relate to heterogeneity in antipsychotic response in schizophrenia, and some studies have shown that glutamatergic metabolite levels in the MFC, thalamus, DLPFC and striatum associate with the degree of antipsychotic response [15–24, 68, 73, 74]. Our findings are relevant to the on-going effort to develop novel drug therapies to target glutamate dysfunction in schizophrenia, as the presence of glutamatergic heterogeneity may indicate the importance of targeting more specific patient subgroups.

### Supplementary information

Supplemental Material
