# Supplementary material for: Variability and magnitude of brain glutamate levels in schizophrenia: a meta and mega-analysis
Source: Mol Psychiatry. 2023 Feb 17;28(5):2039–48. doi: 10.1038/s41380-023-01991-7 (PMC10575771; doi:10.1038/s41380-023-01991-7)
Supplement: Supplementary file 1 — Supplemental Material [file 41380_2023_1991_MOESM1_ESM.docx]

SUPPLEMENTARY INFORMATION

Contents

[eResults 1 – Studies included 2](#_Toc118734934)

[eAppendix 1: Newcastle Ottawa Quality assessment scale for case control studies 3](#_Toc118734935)

[eTable 1: Raw Data Used in the Meta-Analysis 4](#_Toc118734936)

[*Medial Frontal Cortex* 4](#_Toc118734937)

[*Dorsolateral Prefrontal Cortex* 13](#_Toc118734938)

[*Frontal White Matter* 16](#_Toc118734939)

[*Temporal Lobe* 17](#_Toc118734940)

[*Thalamus* 20](#_Toc118734941)

[*Basal Ganglia* 22](#_Toc118734942)

[eTable 2: Quality assessment of studies included in meta-analysis. 24](#_Toc118734943)

[eFigure 1: PRISMA diagram 28](#_Toc118734944)

[eFigure 2: Correlation between mean glutamatergic metabolite level and standard deviation 29](#_Toc118734945)

[eFigure 3: Forest plot of variation ratio (VR) 30](#_Toc118734946)

[eFigure 4: Meta-regressions for glutamate standardised mean differences 31](#_Toc118734947)

[*Age* 31](#_Toc118734948)

[References 32](#_Toc118734949)

## eResults 1 – Studies included

123 studies were included in the meta-analysis (1,2,11,101–110,12,111–120,13,121–123,14–20,3,21–30,4,31–40,5,41–50,6,51–60,7,61–70,8,71–80,9,81–90,10,91–100).

## eAppendix 1: Newcastle Ottawa Quality assessment scale for case control studies

SELECTION

**1) Is the case definition adequate?**

*a) yes, with independent validation (1 point)*

b) yes, e.g. record linkage or based on self-reports

c) no description

**2) Representativeness of the cases**

*a) consecutive or obviously representative series of cases (1 point)*

b) potential for selection biases or not stated

**3) Selection of Controls**

*a) community controls (1 point)*

b) hospital controls

c) no description

**4) Definition of Controls**

*a) no history of disease (1 point)*

b) no description of source

COMPARABILITY

**1) Comparability of cases and controls based on the design or analysis**

*a) study controls for age (1 point)*

EXPOSURE

**1) Ascertainment of exposure**

*a) secure record (1 point)*

*b) structured interview where blind to case/control status (1 point)*

c) interview not blinded to case/control status

d) written self-report or medical record only

e) no description

**2) Same method of ascertainment for cases and controls**

*a) yes (1 point)*

b) no

## eTable 1: Raw Data Used in the Meta-Analysis

Table 1 Glu; glutamate, Gln; glutamine, FEP; first episode psychosis, HV; Healthy Volunteer, PANSS; Positive and Negative Syndrome Scale, CPZ; Chlorpromazine equivalent dose, SD; Standard Deviation. HV sample size was divided by number of clinical groups, or number of brain regions.

| *Medial Frontal Cortex* | | | | | | | | | | | | | | | |
| --- | --- | --- | --- | --- | --- | --- | --- | --- | --- | --- | --- | --- | --- | --- | --- |
| Author | Year |  | Patient (n) | Metabolite Mean | SD | HV (n) | Metabolite Mean | SD | Illness Stage | Med | Mean Age | % Male | PANSS Total | CPZ | Tesla |
| Bartha | 1997 | Glu | 10 | 10.4 | 1.9 | 10.0 | 11.4 | 2.7 | FEP | Naive | 25.4 | 80.0 |  |  | 1.5 |
| Bojesen | 2021 | Glu | 48 | 10.6 | 1.4 | 51.0 | 10.9 | 1.2 | FEP | Naive | 22.4 | 43.0 | 75.9 |  | 3.0 |
| Borgan | 2019 | Glu | 28 | 15.1 | 2.0 | 33.0 | 15.3 | 2.3 | FEP | Med | 26.6 | 77.0 | 89.9 |  | 3.0 |
| Brandt | 2016 | Glu | 24 | 9.2 | 1.7 | 24.0 | 9.1 | 0.7 | Chronic | Med | 37.0 | 81.2 |  |  | 7.0 |
| Bryll | 2020 | Glu | 40 | 0.4 | 0.2 | 30.0 | 0.4 | 0.1 | Chronic | Med | 26.1 | 49.2 | 106.3 |  | 1.5 |
| Bustillo | 2010 | Glu | 14 | 14.6 | 1.4 | 8.0 | 15.8 | 2.6 | FEP | Naive | 28.0 | 83.3 |  |  | 4.0 |
| Bustillo | 2014 | Glu | 84 | 14.1 | 2.6 | 81.0 | 14.1 | 1.4 | Chronic | Med | 36.0 | 75.8 | 62.2 |  | 3.0 |
| Cai | 2022 | Glu | 50 | 17.9 | 2.2 | 43.0 | 17.7 | 2.17 | Chronic | Med | 25.25 | 48.9 | 61.4 | 482.5 | 3.0 |
| Chen | 2017 | Glu | 12 | 5.1 | 2.4 | 12.0 | 3.5 | 1.9 | FEP | Naive | 27.7 | 41.7 | 82.8 |  | 3.0 |
| Chen | 2017 | Glu | 12 | 6.1 | 2.5 | 12.0 | 6.5 | 2.0 | FEP | Naive | 27.7 | 41.7 | 82.8 |  | 3.0 |
| Corcoran | 2020 | Glu | 20 | 7.4 | 1.8 | 28.0 | 7.8 | 1.7 | Chronic | Med | 38.3 | 63.4 | 55.1 |  | 3.0 |
| Demjaha | 2014 | Glu | 8 | 8.9 | 2.4 | 5.0 | 8.6 | 1.0 | Chronic | Med | 45.2 | 55.6 | 50.1 |  | 3.0 |
| Demjaha | 2014 | Glu | 6 | 10.3 | 1.4 | 5.0 | 8.6 | 1.0 | Chronic | Med | 43.8 | 43.8 | 103.7 |  | 3.0 |
| Dempster | 2020 | Glu | 26 | 8.5 | 2.0 | 27.0 | 8.3 | 2.3 | FEP | Med | 22.8 | 71.9 |  |  | 7.0 |
| Egerton | 2018 | Glu | 26 | 1.3 | 0.2 | 36.0 | 1.3 | 0.1 | FEP | Med | 27.9 | 74.2 | 65.4 |  | 3.0 |
| Egerton | 2018 | Glu | 31 | 1.4 | 0.2 | 15.0 | 1.4 | 0.1 | FEP | Med | 22.9 | 52.2 | 83.2 |  | 3.0 |
| Egerton | 2018 | Glu | 13 | 1.3 | 0.1 | 9.0 | 1.3 | 0.1 | FEP | Med | 25.3 | 81.8 | 70.4 |  | 3.0 |
| Falkenberg | 2014 | Glu | 8 | 1.4 | 0.1 | 8.5 | 1.6 | 0.2 | Chronic | Med | 29.0 | 58.8 | 64.0 |  | 3.0 |
| Falkenberg | 2014 | Glu | 8 | 1.6 | 0.2 | 8.5 | 1.7 | 0.2 | Chronic | Med | 29.0 | 58.8 | 64.0 |  | 3.0 |
| Gallinat | 2016 | Glu | 29 | 14.5 | 1.6 | 29.0 | 15.2 | 1.1 | Chronic | Med | 29.2 | 65.5 | 72.7 |  | 3.0 |
| Godlewska | 2021 | Glu | 14 | 11.5 | 0.3 | 18.0 | 12.2 | 0.2 | FEP | Med | 26.4 | 100.0 | 58.0 |  | 7.0 |
| Goldstein | 2015 | Glu | 14 | 1.9 | 0.1 | 4.3 | 2.0 | 0.1 | Chronic | Med | 32.4 | 80.3 | 59.9 | 426.5 | 3.0 |
| Goldstein | 2015 | Glu | 14 | 1.8 | 0.1 | 4.3 | 2.0 | 0.1 | Chronic | Med | 33.9 | 76.6 | 59.7 | 446.0 | 3.0 |
| Goldstein | 2015 | Glu | 9 | 1.9 | 0.1 | 4.3 | 2.0 | 0.1 | Chronic | Med | 34.5 | 81.6 | 62.4 | 855.4 | 3.0 |
| Korenic | 2020 | Glu | 19 | 13.2 | 1.2 | 22.0 | 13.3 | 1.3 | Chronic | Med | 29.9 | 63.4 |  |  | 3.0 |
| Kumar | 2020 | Glu | 27 | 6.0 | 0.7 | 45.0 | 6.2 | 0.8 | Chronic | Med | 27.5 | 67.9 |  |  | 7.0 |
| Hjelmervik | 2020 | Glu | 33 | 17.1 | 1.8 | 33.0 | 16.6 | 1.7 | Chronic | Med | 30.0 |  | 62.5 |  | 3.0 |
| Huang ACC | 2022 | Glu* | 17.5 | 1.1 | 0.2 | 4.8 | 1.1 | 0.2 | Chronic | Med | 38.91 | 48.0 | 38.2 | 467.9 | 1.5 |
| Huang ACC | 2022 | Glu* | 19 | 1.3 | 0.4 | 4.8 | 1.1 | 0.2 | Chronic | Med | 40.8 | 51.3 | 83.2 | 821.4 | 1.5 |
| Huang PFC | 2022 | Glu* | 17.5 | 1.5 | 0.4 | 4.8 | 1.1 | 0.2 | Chronic | Med | 38.9 | 47.8 | 38.2 | 467.9 | 1.5 |
| Huang PFC | 2022 | Glu* | 19 | 1.6 | 0.8 | 4.8 | 1.1 | 0.2 | Chronic | Med | 40.9 | 51.3 | 83.21 | 821.4 | 1.5 |
| Iwata | 2019 | Glu | 26 | 17.2 | 2.0 | 8.7 | 16.0 | 1.7 | Chronic | Med | 43.0 | 77.0 | 82.8 | 643.2 | 3.0 |
| Iwata | 2019 | Glu | 27 | 16.9 | 1.4 | 8.7 | 16.0 | 1.7 | Chronic | Med | 40.6 | 71.8 | 56.1 | 527.1 | 3.0 |
| Iwata | 2019 | Glu | 21 | 16.7 | 1.5 | 8.7 | 16.0 | 1.7 | Chronic | Med | 43.5 | 74.7 | 57.2 | 443.1 | 3.0 |
| Jauhar | 2018 | Glu | 26 | 11.6 | 1.8 | 20.0 | 11.8 | 1.6 | FEP | Med | 24.4 | 72.0 | 69.2 |  | 3.0 |
| Jeon | 2021 | Glu | 21 | 6.5 | 0.6 | 10.0 | 7.25 | 1.3 | FEP | Naïve | 22.0 | 67.7 |  |  | 7.0 |
| Kim | 2018 | Glu | 40 | 1.4 | 0.2 | 50.0 | 1.2 | 0.2 | FEP | Med | 24.4 | 58.8 | 47.3 | 127.3 | 4.0 |
| Legind | 2019 | Glu | 28 | 10.4 | 1.1 | 49.0 | 10.4 | 1.0 | Chronic | Med | 39.6 | 58.4 | 62.9 |  | 3.0 |
| Legind | 2019 | Glu | 22 | 10.4 | 1.6 | 36.0 | 10.3 | 0.9 | Chronic | Med | 42.2 | 56.9 | 60.6 |  | 3.0 |
| Li | 2020 | Glu | 35 | 8.9 | 1.1 | 40.0 | 8.8 | 0.9 | FEP | Naive | 22.5 | 62.7 |  |  | 3.0 |
| Lutkenhoff | 2010 | Glu | 9 | 8.1 | 3.3 | 21.0 | 11.8 | 3.8 | Chronic | Med | 52.2 | 56.7 |  |  | 3.0 |
| Marsman | 2014 | Glu | 14 | 8.5 | 1.3 | 18.0 | 8.6 | 1.1 | Chronic | Med | 27.6 | 72.5 | 53.1 |  | 7.0 |
| Ongur | 2008 | Glu | 17 | 0.9 | 0.3 | 21.0 | 1.1 | 0.3 | Chronic | Med | 38.0 | 55.3 | 86.0 |  | 4.0 |
| Ongur | 2010 | Glu | 21 | 0.9 | 0.2 | 19.0 | 0.8 | 0.2 | Chronic | Med | 37.6 | 65.0 | 50.5 | 555.0 | 4.0 |
| Onwordi | 2021 | Glu | 18 | 1.1 | 0.0 | 22.0 | 1.1 | 0.0 | Chronic | Med | 39.6 | 90.0 |  |  | 3.0 |
| Posporelis | 2018 | Glu | 20 | 1.3 | 0.1 | 20.0 | 1.3 | 0.1 | FEP | Med | 23.7 | 65.0 |  | 188.0 | 7.0 |
| Reid | 2018 | Glu | 21 | 6.6 | 0.5 | 21.0 | 6.9 | 0.5 | FEP | Med | 23.4 | 76.2 |  |  | 7.0 |
| Roalf | 2017 | Glu | 5 | 5.1 | 0.3 | 17.0 | 5.2 | 0.3 | FEP | Med | 20.7 | 54.5 |  |  | 7.0 |
| Rowland | 2016b | Glu | 27 | 7.9 | 0.8 | 29.0 | 8.1 | 0.7 | Chronic | Med | 32.0 | 55.4 |  | 381.1 | 7.0 |
| Rowland | 2016 | Glu | 45 | 8.6 | 1.0 | 53.0 | 9.2 | 0.8 | Chronic | Med | 37.4 | 62.2 |  |  | 3.0 |
| Shirayama | 2010 | Glu | 19 | 9.4 | 1.5 | 18.0 | 9.6 | 1.0 | Chronic | Med | 30.9 | 70.3 |  | 267.0 | 3.0 |
| Smesny | 2015 | Glu | 31 | 10.5 | 0.8 | 31.0 | 9.8 | 0.5 | FEP | Naive | 25.7 | 50.0 | 58.7 |  | 3.0 |
| Smesny | 2021 | Glu | 29 | 8.4 | 1.0 | 26.0 | 8.9 | 0.7 | FEP | Naive | 25.5 | 47.6 |  |  | 3.0 |
| Taylor | 2017 | Glu | 16 | 10.7 | 1.2 | 18.0 | 10.0 | 1.3 | Chronic | Med | 23.3 | 70.6 |  | 358.0 | 7.0 |
| Tayoshi | 2009 | Glu | 30 | 9.8 | 2.7 | 25.0 | 11.5 | 3.6 | Chronic | Med | 34.4 | 49.1 | 55.2 |  | 3.0 |
| Terpstra | 2005 | Glu | 13 | 10.2 | 0.9 | 9.0 | 10.0 | 0.7 | Chronic | Med | 25.5 | 54.5 |  |  | 4.0 |
| Theberge | 2002 | Glu | 20 | 16.0 | 2.3 | 20.0 | 16.4 | 2.3 | FEP | Naive | 26.0 | 70.0 |  |  | 4.0 |
| Theberge | 2003 | Glu | 21 | 13.7 | 3.4 | 21.0 | 15.9 | 2.8 | Chronic | Med | 35.0 | 95.2 |  |  | 4.0 |
| Tibbo | 2013 | Glu | 33 | 8.1 | 1.9 | 41.0 | 7.8 | 1.8 | FEP | Naive | 21.8 | 79.7 | 75.5 |  | 3.0 |
| Wang | 2019 | Glu | 75 | 7.8 | 0.6 | 87.0 | 8.2 | 0.6 | FEP | Med | 22.8 | 58.3 |  | 188.0 | 7.0 |
| Wijtenburg | 2017 | Glu | 48 | 9.2 | 0.8 | 54.0 | 9.5 | 0.7 | Chronic | Med | 25.2 | 57.8 |  |  | 3.0 |
| Wijtenburg | 2017 | Glu | 47 | 8.2 | 1.0 | 39.0 | 8.7 | 0.8 | Chronic | Med | 50.4 | 59.3 |  |  | 3.0 |
| Wijtenburg | 2021 | Glu | 39 | 8.8 | 0.8 | 37.0 | 9.1 | 0.9 | Chronic | Med | 32.4 | 53.8 |  | 336.7 | 7.0 |
| Xin | 2016 | Glu | 25 | 13.5 | 1.6 | 33.0 | 14.2 | 1.0 | FEP | Med | 25.1 | 62.1 |  | 325.0 | 3.0 |
| Yang | 2015 | Glu | 22 | 2.8 | 1.4 | 23.0 | 2.3 | 1.1 | FEP | Naive | 25.8 | 42.2 | 69.2 |  | 3.0 |
| Bartha | 1997 | Gln | 10 | 7.2 | 3.7 | 10.0 | 3.8 | 2.0 | FEP | Naive | 25.4 | 80.0 |  |  | 1.5 |
| Brandt | 2016 | Gln | 24 | 2.3 | 0.6 | 24.0 | 2.4 | 0.5 | Chronic | Med | 37.0 | 81.2 |  |  | 7.0 |
| Bustillo | 2010 | Gln | 14 | 10.3 | 3.1 | 8.0 | 7.9 | 1.8 | FEP | Naive | 28.0 | 83.3 |  |  | 4.0 |
| Bustillo | 2014 | Gln | 72 | 4.7 | 2.5 | 76.0 | 3.8 | 1.8 | Chronic | Med | 36.0 | 75.8 | 62.2 |  | 3.0 |
| Chen | 2017 | Gln | 24 | 2.5 | 1.7 | 24.0 | 4.4 | 1.9 | FEP | Naive | 27.7 | 41.7 | 82.8 |  | 3.0 |
| Bojesen | 2021 | Gln | 40 | 3.7 | 0.8 | 40.0 | 3.6 | 0.6 | FEP | Naive | 22.4 | 43.0 | 75.9 |  | 3.0 |
| Godlewska | 2021 | Gln | 14 | 3.4 | 0.2 | 18.0 | 3.9 | 0.1 | FEP | Med | 26.4 | 100.0 | 58.0 |  | 7.0 |
| Hjelmervik | 2020 | Gln | 33 | 4.2 | 2.4 | 33.0 | 4.3 | 2.3 | Chronic | Med | 30.0 |  | 62.5 |  | 3.0 |
| Jessen | 2013 | Gln | 20 | 0.3 | 0.1 | 20.0 | 0.2 | 0.1 | Chronic | Med | 32.6 | 62.5 | 51.0 |  | 3.0 |
| Jeon | 2021 | Gln | 21 | 1.1 | 0.3 | 10 | 1.1 | 0.4 | FEP | Naïve | 22.0 | 67.7 |  |  | 7.0 |
| Kumar | 2020 | Gln | 27 | 1.5 | 0.4 | 42.0 | 1.7 | 0.3 | Chronic | Med | 27.5 | 67.9 |  |  | 7.0 |
| Ongur | 2008 | Gln | 17 | 0.4 | 0.3 | 21.0 | 0.4 | 0.2 | Chronic | Med | 38.0 | 55.3 | 86.0 |  | 4.0 |
| Reid | 2018 | Gln | 21 | 1.8 | 0.3 | 21.0 | 1.9 | 0.2 | FEP | Med | 23.4 | 76.2 |  |  | 7.0 |
| Rowland | 2016b | Gln | 27 | 1.9 | 0.3 | 29.0 | 1.8 | 0.4 | Chronic | Med | 32.0 | 55.4 |  | 381.1 | 7.0 |
| Rowland | 2016 | Gln | 45 | 2.2 | 0.4 | 53.0 | 2.3 | 0.4 | Chronic | Med | 37.4 | 62.2 |  |  | 3.0 |
| Shirayama | 2010 | Gln | 19 | 3.4 | 0.7 | 18.0 | 3.0 | 0.7 | Chronic | Med | 30.9 | 70.3 |  | 267.0 | 3.0 |
| Taylor | 2017 | Gln | 16 | 2.0 | 1.0 | 18.0 | 2.0 | 0.5 | Chronic | Med | 23.3 | 70.6 |  | 358.0 | 7.0 |
| Tayoshi | 2009 | Gln | 30 | 4.9 | 1.8 | 25.0 | 5.6 | 2.3 | Chronic | Med | 34.4 | 49.1 | 55.2 |  | 3.0 |
| Terpstra | 2005 | Gln | 12 | 3.1 | 0.8 | 8.0 | 3.1 | 0.7 | Chronic | Med | 25.5 | 54.0 |  |  | 4.0 |
| Theberge | 2002 | Gln | 20 | 8.7 | 1.8 | 20.0 | 7.4 | 1.6 | FEP | Naive | 26.0 | 66.7 |  |  | 4.0 |
| Theberge | 2003 | Gln | 21 | 7.0 | 2.1 | 21.0 | 8.8 | 2.8 | Chronic | Med | 35.0 | 95.2 |  |  | 4.0 |
| Wang | 2019 | Gln | 74 | 1.8 | 0.3 | 89.0 | 1.8 | 0.3 | FEP | Med | 22.8 | 58.3 |  | 188.0 | 7.0 |
| Wijtenburg | 2017 | Gln | 48 | 2.3 | 0.5 | 54.0 | 2.2 | 0.4 | Chronic | Med | 25.2 | 57.8 |  |  | 3.0 |
| Wijtenburg | 2017 | Gln | 47 | 2.6 | 1.0 | 39.0 | 2.4 | 0.5 | Chronic | Med | 50.4 | 59.3 |  |  | 3.0 |
| Wijtenburg | 2021 | Gln | 39 | 2.1 | 0.4 | 37.0 | 2.0 | 0.4 | Chronic | Med | 32.4 | 53.8 |  | 336.7 | 7.0 |
| Yang | 2015 | Gln | 22 | 4.1 | 2.6 | 23.0 | 3.7 | 1.4 | FEP | Naive | 25.8 | 42.2 | 69.2 |  | 3.0 |
| Birur | 2020 | Glx | 20 | 0.2 | 0.1 | 18.0 | 0.2 | 0.0 | FEP | Naive | 22.7 | 63.0 |  |  | 3.0 |
| Bustillo | 2021 | Glx | 48 | 17.0 | 3.0 | 51.0 | 16.2 | 2.6 | FEP | Med | 23.1 | 63.6 |  | 126.0 | 3.0 |
| Batalla | 2015 | Glx | 10 | 7.8 | 1.1 | 6.0 | 9.2 | 1.1 | FEP | Naive | 23.1 | 50.0 | 117.4 |  | 3.0 |
| Egerton | 2018 | Glx | 26 | 1.9 | 0.3 | 36.0 | 1.9 | 0.2 | FEP | Med | 27.9 | 74.2 | 65.4 |  | 3.0 |
| Egerton | 2018 | Glx | 31 | 1.6 | 0.3 | 15.0 | 1.5 | 0.2 | FEP | Med | 22.9 | 52.2 | 83.2 |  | 3.0 |
| Egerton | 2018 | Glx | 13 | 1.5 | 0.2 | 9.0 | 1.6 | 0.2 | FEP | Med | 25.3 | 81.8 | 70.4 |  | 3.0 |
| Bartolomeo | 2019 | Glx | 34 | 4.9 | 1.4 | 19.0 | 4.1 | 1.2 | FEP | Med | 22.4 | 54.7 | 53.7 | 139.6 | 3.0 |
| Bojesen | 2021 | Glx | 37 | 10.4 | 1.6 | 47.0 | 11.0 | 1.2 | FEP | Naive | 22.4 | 43.0 | 75.9 |  | 3.0 |
| Borgan | 2019 | Glx | 28 | 19.7 | 3.2 | 33.0 | 19.8 | 4.1 | FEP | Med | 26.7 | 77.0 | 89.9 |  | 3.0 |
| Cen | 2020 | Glx | 23 | 5.3 | 0.9 | 26.0 | 5.0 | 0.7 | FEP | Naive | 26.4 | 40.8 | 76.0 |  | 3.0 |
| Chiu | 2017 | Glx | 19 | 12.9 | 3.2 | 14.0 | 7.9 | 1.4 | FEP | Med | 28.4 | 60.6 | 47.5 | 389.5 | 3.0 |
| Coughlin | 2015 | Glx | 25 | 1.5 | 0.2 | 17.0 | 1.5 | 0.2 | Chronic | Med | 33.1 | 79.2 |  |  | 3.0 |
| Coughlin | 2020 | Glx | 46 | 1.5 | 0.2 | 50.0 | 1.6 | 0.2 | Chronic | Med | 33.1 | 70.8 |  | 381.3 | 3.0 |
| de la Fuente-Sandoval | 2018 | Glx | 28 | 1.9 | 0.3 | 18.0 | 1.7 | 0.3 | FEP | Naive | 23.0 | 63.0 | 104.4 |  | 3.0 |
| Demjaha | 2014 | Glx | 8 | 13.3 | 2.8 | 5.0 | 12.2 | 1.6 | Chronic | Med | 45.2 | 55.6 | 50.1 |  | 3.0 |
| Demjaha | 2014 | Glx | 6 | 13.8 | 2.9 | 5.0 | 12.2 | 1.6 | Chronic | Med | 43.8 | 43.8 | 103.7 |  | 3.0 |
| Goto | 2012 | Glx | 18 | 0.8 | 0.3 | 18.0 | 0.7 | 0.4 | FEP | Med | 30.0 | 50.0 | 68.1 |  | 3.0 |
| Goldstein | 2015 | Glx | 14 | 2.3 | 0.2 | 4.3 | 2.4 | 0.2 | Chronic | Med | 32.4 | 80.3 | 59.9 | 426.5 | 3.0 |
| Goldstein | 2015 | Glx | 14 | 2.1 | 0.2 | 4.3 | 2.4 | 0.2 | Chronic | Med | 33.9 | 76.6 | 59.7 | 446.0 | 3.0 |
| Goldstein | 2015 | Glx | 9 | 2.3 | 0.2 | 4.3 | 2.4 | 0.2 | Chronic | Med | 34.5 | 81.6 | 62.4 | 855.4 | 3.0 |
| Hjelmervik | 2020 | Glx | 38 | 20.2 | 3.3 | 37.0 | 19.8 | 3.6 | Chronic | Med | 30.0 |  | 62.5 |  | 3.0 |
| Iwata | 2019 | Glx | 26 | 21.9 | 3.1 | 8.7 | 20.1 | 2.1 | Chronic | Med | 43.0 | 77.0 | 82.8 | 643.2 | 3.0 |
| Iwata | 2019 | Glx | 27 | 21.3 | 1.6 | 8.7 | 20.1 | 2.1 | Chronic | Med | 40.6 | 71.8 | 56.1 | 527.1 | 3.0 |
| Iwata | 2019 | Glx | 21 | 20.7 | 2.5 | 8.7 | 20.1 | 2.1 | Chronic | Med | 43.5 | 74.7 | 57.2 | 443.1 | 3.0 |
| Jauhar | 2018 | Glx | 26 | 17.2 | 2.3 | 20.0 | 17.6 | 2.0 | FEP | Med | 24.4 | 72.0 | 69.2 |  | 3.0 |
| Jessen | 2013 | Glx | 20 | 2.1 | 0.3 | 20.0 | 2.1 | 0.3 | Chronic | Med | 32.6 | 62.5 | 51.0 |  | 3.0 |
| Kegeles | 2012 | Glx | 16 | 0.2 | 0.0 | 11.0 | 0.1 | 0.0 | Chronic | Med | 32.5 | 66.6 | 71.0 |  | 3.0 |
| Kegeles | 2012 | Glx | 16 | 0.1 | 0.0 | 11.0 | 0.1 | 0.0 | Chronic | Med | 32.5 | 66.6 | 57.0 |  | 3.0 |
| Kraguljac | 2012 | Glx | 46 | 0.7 | 0.1 | 46.0 | 0.7 | 0.1 | Chronic | Med | 37.1 | 68.0 | 58.0 |  | 3.0 |
| Kraguljac | 2019 | Glx | 61 | 0.7 | 0.1 | 31.0 | 0.7 | 0.1 | Chronic | Med | 28.2 | 71.5 |  |  | 3.0 |
| Legind | 2019 | Glx | 28 | 13.5 | 1.5 | 49.0 | 13.8 | 1.6 | Chronic | Med | 39.6 | 58.4 | 62.9 |  | 3.0 |
| Legind | 2019 | Glx | 22 | 13.7 | 2.4 | 36.0 | 13.8 | 1.4 | Chronic | Med | 42.2 | 56.9 | 60.6 |  | 3.0 |
| Leptourgos | 2022 | Glx | 35 | 15.3 | 0.9 | 12.0 | 15.5 | 1.1 | Chronic | Med | 34.6 | 63.0 |  | 331.8 | 3.0 |
| Liemburg | 2016 | Glx | 31 | 10.9 | 1.5 | 18.0 | 10.9 | 1.2 | FEP | Med | 26.8 | 68.7 | 62.5 |  | 3.0 |
| Liemburg | 2016 | Glx | 60 | 10.0 | 1.6 | 18.0 | 10.9 | 1.2 | Chronic | Med | 32.4 | 75.0 | 62.4 |  | 3.0 |
| Matrone | 2022 | Glx | 10 | 1.0 | 0.9 | 10 | 0.7 | 0.5 | Chronic | Med |  | 50.0 |  |  | 1.5 |
| Matrone | 2022 | Glx | 10 | 0.9 | 0.9 | 10 | 0.7 | 0.5 | Chronic | Med |  | 80.0 |  |  | 1.5 |
| Natsubori | 2014 | Glx | 19 | 12.4 | 2.4 | 19.0 | 12.4 | 2.3 | FEP | Med | 25.9 | 73.7 | 71.3 |  | 3.0 |
| Natsubori | 2014 | Glx | 25 | 11.0 | 2.8 | 28.0 | 12.6 | 2.5 | Chronic | Med | 32.8 | 60.4 | 76.1 |  | 3.0 |
| Ohrmann | 2008 | Glx | 43 | 8.3 | 3.3 | 37.0 | 7.7 | 2.2 | Chronic | Med | 27.6 | 71.2 | 65.1 |  | 1.5 |
| Onwordi | 2021 | Glx | 18 | 1.3 | 0.0 | 22.0 | 1.3 | 0.0 | Chronic | Med | 39.6 | 90.0 |  |  | 3.0 |
| Pillinger | 2019 | Glx | 19 | 22.7 | 7.0 | 18.0 | 20.4 | 4.2 | Chronic | Med | 38.0 | 83.8 | 72.5 |  | 3.0 |
| Rowland | 2009 | Glx | 9 | 9.6 | 2.0 | 5.5 | 10.7 | 2.5 | Chronic | Med | 38.5 | 76.2 | 61.0 |  | 3.0 |
| Rowland | 2009 | Glx | 9 | 11.1 | 2.4 | 5.5 | 10.7 | 2.5 | Chronic | Med | 40.0 | 76.2 | 66.0 |  | 3.0 |
| Rowland | 2013 | Glx | 11 | 9.3 | 2.4 | 10.0 | 10.0 | 1.1 | Chronic | Med | 31.8 | 66.7 | 63.0 |  | 3.0 |
| Rowland | 2013 | Glx | 10 | 8.3 | 2.3 | 10.0 | 9.7 | 1.2 | Chronic | Med | 50.2 | 70.0 | 57.0 |  | 3.0 |
| Smesny | 2021 | Glx | 29 | 11.8 | 1.7 | 26.0 | 12.4 | 1.5 | FEP | Naive | 25.5 | 47.6 |  |  | 3.0 |
| Tarumi | 2020 | Glx | 25 | 21.2 | 3.1 | 14.0 | 19.2 | 2.2 | Chronic | Med | 43.8 | 44.0 | 111.2 | 958.5 | 3.0 |
| Tarumi | 2020 | Glx | 26 | 20.7 | 2.1 | 14.0 | 19.2 | 2.2 | Chronic | Med | 43.0 | 45.5 | 49.8 | 412.4 | 3.0 |
| Thomas | 1998 | Glx | 12 | 1.1 | 0.3 | 12.0 | 1.2 | 0.4 | FEP | Naive | 12.5 | 54.2 |  |  | 1.5 |
| Wang | 2016 | Glx | 16 | 12.8 | 2.7 | 23.0 | 16.6 | 3.2 | FEP | Naive | 22.3 | 48.7 |  |  | 3.0 |
| Wang | 2019 | Glx | 75 | 9.6 | 0.8 | 88.0 | 9.9 | 0.8 | FEP | Med | 22.8 | 58.3 |  | 188.0 | 7.0 |
| Wang | 2022 | Glx | 114 | 8.0 | 2.2 | 59.0 | 8.27 | 1.8 | Chronic | Med | 26.7 | 65.3 | 56.0 | 667.9 | 3.0 |
| Wijtenburg | 2017 | Glx | 48 | 11.5 | 1.1 | 54.0 | 11.7 | 1.0 | Chronic | Med | 25.2 | 57.8 |  |  | 3.0 |
| Wijtenburg | 2017 | Glx | 47 | 10.6 | 1.4 | 39.0 | 11.0 | 1.1 | Chronic | Med | 50.4 | 59.3 |  |  | 3.0 |
| Wood | 2007 | Glx | 15 | 9.9 | 1.1 | 14.0 | 9.5 | 2.0 | Chronic | Med | 32.5 | 100.0 | 58.1 |  | 3.0 |

*Glu*= Glu/NAA ratio*

| *Dorsolateral Prefrontal Cortex* | | | | | | | | | | | | | | | |
| --- | --- | --- | --- | --- | --- | --- | --- | --- | --- | --- | --- | --- | --- | --- | --- |
| Author | Year |  | Patient (n) | Metabolite Mean | SD | HV (n) | Metabolite Mean | SD | Illness Stage | Med | Mean Age | % Male | PANSS Total | CPZ | Tesla |
| Corcoran | 2020 | Glu | 20 | 7.4 | 1.2 | 34.0 | 8.2 | 1.4 | Chronic | Med | 39.5 | 63.4 | 55.1 |  | 3.0 |
| Da Silva | 2011 | Glu | 11 | 6.4 | 1.3 | 23.0 | 6.4 | 1.4 | Chronic | Med | 30.2 | 58.8 | 59.0 |  | 3.0 |
| Godlewska | 2021 | Glu | 14 | 8.9 | 0.2 | 16.0 | 9.0 | 0.6 | FEP | Med | 26.4 | 100.0 | 58.0 |  | 7.0 |
| Goldstein | 2015 | Glu | 15 | 1.2 | 0.0 | 5.3 | 1.1 | 0.0 | Chronic | Med | 32.4 | 80.3 | 59.9 | 426.5 | 3.0 |
| Goldstein | 2015 | Glu | 16 | 1.2 | 0.0 | 5.3 | 1.1 | 0.0 | Chronic | Med | 33.9 | 76.6 | 59.7 | 446.0 | 3.0 |
| Goldstein | 2015 | Glu | 11 | 1.1 | 0.1 | 5.3 | 1.1 | 0.0 | Chronic | Med | 34.5 | 81.6 | 62.4 | 855.4 | 3.0 |
| Iwata | 2019 | Glu | 21 | 13.6 | 1.6 | 8.7 | 13.9 | 1.3 | Chronic | Med | 43.0 | 77.0 | 82.8 | 643.2 | 3.0 |
| Iwata | 2019 | Glu | 22 | 13.9 | 1.6 | 8.7 | 13.9 | 1.3 | Chronic | Med | 40.6 | 71.8 | 56.1 | 527.1 | 3.0 |
| Iwata | 2019 | Glu | 21 | 14.6 | 1.3 | 8.7 | 13.9 | 1.3 | Chronic | Med | 43.5 | 74.7 | 57.2 | 443.1 | 3.0 |
| Kaminski | 2020 | Glu | 36 | 7.9 | 1.2 | 17.5 | 8.2 | 0.9 | Chronic | Med | 35.0 | 72.0 | 87.0 | 364.3 | 3.0 |
| Kaminski | 2020 | Glu | 19 | 7.9 | 0.8 | 17.5 | 8.2 | 0.9 | Chronic | Med | 33.7 | 70.2 | 108.7 |  | 3.0 |
| Lesh | 2021 | Glu | 33 | 1.2 | 0.1 | 38.0 | 1.2 | 0.1 | FEP | Med | 21.7 | 68.4 |  | 226.6 | 3.0 |
| Ragland | 2020 | Glu | 38 | 0.9 | 0.1 | 49.0 | 0.9 | 0.1 | FEP | Med | 23.9 | 68.5 |  |  | 3.0 |
| Smesny | 2015 | Glu | 31 | 9.1 | 0.7 | 31.0 | 8.5 | 1.1 | FEP | Naive | 25.7 | 50.0 | 58.7 |  | 3.0 |
| Smesny | 2021 | Glu | 29 | 9.8 | 1.4 | 26.0 | 10.9 | 1.6 | FEP | Naive | 25.5 | 47.6 |  |  | 3.0 |
| Smucny | 2022 | Glu | 37 | 6.7 | 0.1 | 42.0 | 6.8 | 0.1 | FEP | Med | 20.9 | 67.8 |  | 225.1 | 3.0 |
| Stanley | 1996 | Glu | 13 | 9.7 | 2.3 | 8.0 | 9.0 | 1.9 | FEP | Naive | 29.0 | 94.6 |  |  | 1.5 |
| Stanley | 1996 | Glu | 12 | 10.4 | 2.1 | 8.0 | 9.0 | 1.9 | FEP | Med | 29.0 | 94.4 |  |  | 1.5 |
| Stanley | 1996 | Glu | 12 | 7.9 | 1.5 | 8.0 | 9.0 | 1.9 | Chronic | Med | 36.5 | 97.2 |  |  | 1.5 |
| Stanley | 2007 | Glu | 18 | 6.2 | 1.0 | 61.0 | 6.7 | 1.4 | FEP | Naive | 23.7 | 65.8 | 93.0 |  | 1.5 |
| Wang | 2019 | Glu | 72 | 6.4 | 0.8 | 84.0 | 6.6 | 0.5 | FEP | Med | 22.8 | 58.3 |  | 188.0 | 7.0 |
| Wijtenburg | 2021 | Glu | 38 | 7.5 | 0.1 | 38.0 | 7.3 | 0.8 | Chronic | Med | 32.4 | 53.8 |  | 336.7 | 7.0 |
| Da Silva | 2011 | Gln | 11 | 3.2 | 1.4 | 23.0 | 2.9 | 0.9 | Chronic | Med | 30.2 | 58.8 | 59.0 |  | 3.0 |
| Godlewska | 2021 | Gln | 14 | 2.5 | 0.1 | 16.0 | 2.8 | 0.1 | FEP | Med | 26.4 | 100.0 | 58.0 |  | 7.0 |
| Jessen | 2013 | Gln | 20 | 0.2 | 0.1 | 20.0 | 0.2 | 0.0 | Chronic | Med | 32.6 | 62.5 | 51.0 |  | 3.0 |
| Stanley | 1996 | Gln | 13 | 4.4 | 2.0 | 8.0 | 5.1 | 1.7 | FEP | Naive | 29.0 | 94.6 |  |  | 1.5 |
| Stanley | 1996 | Gln | 12 | 4.4 | 2.6 | 8.0 | 5.1 | 1.7 | FEP | Med | 29.0 | 94.4 |  |  | 1.5 |
| Stanley | 1996 | Gln | 12 | 6.6 | 1.9 | 8.0 | 5.1 | 1.7 | Chronic | Med | 36.5 | 97.2 |  |  | 1.5 |
| Wang | 2019 | Gln | 68 | 1.4 | 0.3 | 77.0 | 1.4 | 0.3 | FEP | Med | 22.8 | 58.3 |  | 188.0 | 7.0 |
| Wijtenburg | 2021 | Gln | 38 | 1.6 | 0.4 | 36.0 | 1.4 | 0.3 | Chronic | Med | 32.4 | 53.8 |  | 336.7 | 7.0 |
| Block | 2000 | Glx | 25 | 1.8 | 0.6 | 19.0 | 1.9 | 0.2 | Chronic | Med | 37.9 | 56.8 |  |  | 1.5 |
| Coughlin | 2015 | Glx | 25 | 1.4 | 0.2 | 16.0 | 1.4 | 0.2 | Chronic | Med | 33.1 | 79.2 |  |  | 3.0 |
| Da Silva | 2011 | Glx | 11 | 9.6 | 2.3 | 23.0 | 9.2 | 2.1 | Chronic | Med | 30.2 | 58.8 | 59.0 |  | 3.0 |
| Goldstein | 2015 | Glx | 15 | 1.5 | 0.1 | 5.3 | 1.3 | 0.1 | Chronic | Med | 32.4 | 80.3 | 59.9 | 426.5 | 3.0 |
| Goldstein | 2015 | Glx | 16 | 1.4 | 0.1 | 5.3 | 1.3 | 0.1 | Chronic | Med | 33.9 | 76.6 | 59.7 | 446.0 | 3.0 |
| Goldstein | 2015 | Glx | 11 | 1.2 | 0.1 | 5.3 | 1.3 | 0.1 | Chronic | Med | 34.5 | 81.6 | 62.4 | 855.4 | 3.0 |
| Huang | 2017 | Glx | 58 | 1.7 | 1.2 | 43.0 | 1.4 | 0.7 | FEP | Naive | 22.9 | 44.6 | 80.9 |  | 3.0 |
| Iwata | 2019 | Glx | 21 | 17.0 | 2.5 | 8.7 | 17.0 | 1.7 | Chronic | Med | 43.0 | 77.0 | 82.8 | 643.2 | 3.0 |
| Iwata | 2019 | Glx | 22 | 17.1 | 2.1 | 8.7 | 17.0 | 1.7 | Chronic | Med | 40.6 | 71.8 | 56.1 | 527.1 | 3.0 |
| Iwata | 2019 | Glx | 21 | 18.1 | 1.8 | 8.7 | 17.0 | 1.7 | Chronic | Med | 43.5 | 74.7 | 57.2 | 443.1 | 3.0 |
| Jessen | 2013 | Glx | 20 | 1.9 | 0.2 | 20.0 | 1.9 | 0.2 | Chronic | Med | 32.6 | 62.5 | 51.0 |  | 3.0 |
| Kegeles | 2012 | Glx | 16 | 0.2 | 0.1 | 11.0 | 0.1 | 0.0 | Chronic | Med | 32.5 | 66.0 | 71.0 |  | 3.0 |
| Kegeles | 2012 | Glx | 16 | 0.2 | 0.1 | 11.0 | 0.1 | 0.0 | Chronic | Med | 32.5 | 66.0 | 57.0 |  | 3.0 |
| Leptourgos | 2022 | Glx | 35 | 11.1 | 1.6 | 12.0 | 11.6 | 1.44 | Chronic | Med | 34.6 | 63.0 |  | 331.8 | 3.0 |
| Ohrmann | 2005 | Glx | 21 | 4.6 | 3.2 | 10.5 | 12.3 | 5.6 | Chronic | Med | 28.9 | 66.7 | 63.0 |  | 1.5 |
| Ohrmann | 2005 | Glx | 18 | 10.0 | 6.3 | 10.5 | 12.3 | 5.6 | FEP | Naive | 28.6 | 66.7 | 85.9 |  | 1.5 |
| Ohrmann | 2007 | Glx | 20 | 4.6 | 3.3 | 10.0 | 12.5 | 5.7 | Chronic | Med | 29.2 | 67.5 | 61.9 |  | 1.5 |
| Ohrmann | 2007 | Glx | 15 | 10.4 | 6.4 | 10.0 | 12.5 | 5.7 | FEP | Naive | 27.6 | 65.7 | 84.6 |  | 1.5 |
| Ohrmann | 2008 | Glx | 43 | 8.3 | 3.0 | 37.0 | 8.0 | 3.0 | Chronic | Med | 27.6 | 71.2 | 65.1 |  | 1.5 |
| Smesny | 2021 | Glx | 29 | 14.9 | 1.5 | 26.0 | 15.2 | 1.6 | FEP | Naive | 25.5 | 47.6 |  |  | 3.0 |
| Wang | 2019 | Glx | 71 | 7.8 | 0.9 | 85.0 | 8.0 | 0.7 | FEP | Med | 22.8 | 58.3 |  | 188.0 | 7.0 |
| Xiang | 2019 | Glx | 20 | 9.6 | 2.5 | 26.0 | 10.9 | 2.6 | FEP | Med | 27.6 | 28.2 | 81.7 | 516.5 | 3.0 |

| *Frontal White Matter* | | | | | | | | | | | | | | | |
| --- | --- | --- | --- | --- | --- | --- | --- | --- | --- | --- | --- | --- | --- | --- | --- |
| Author | Year |  | Patient (n) | Metabolite Mean | SD | HV (n) | Metabolite Mean | SD | Illness Stage | Med | Mean Age | % Male | PANSS Total | CPZ | Tesla |
| Bryant | 2021 | Glu | 35 | 10.9 | 1.4 | 28.5 | 10.1 | 1.4 | FEP | Naive | 24.2 | 58.0 |  |  | 3.0 |
| Bryant | 2021 | Glu | 22 | 10.1 | 1.4 | 28.5 | 10.1 | 1.4 | FEP | Naive | 23.7 | 78.0 |  |  | 3.0 |
| Bustillo | 2010 | Glu | 10 | 8.8 | 2.1 | 10.0 | 9.0 | 1.5 | FEP | Naive | 28.0 | 83.3 |  |  | 4.0 |
| Lutkenhoff | 2010 | Glu | 9 | 4.2 | 1.7 | 21.0 | 5.5 | 2.0 | Chronic | Med | 52.2 | 56.7 |  |  | 3.0 |
| Smesny | 2015 | Glu | 31 | 10.5 | 0.9 | 31.0 | 9.5 | 1.0 | FEP | Naive | 25.7 | 50.0 | 58.7 |  | 3.0 |
| Tunc-Skarka | 2009 | Glu | 17 | 6.8 | 1.1 | 26.0 | 6.5 | 1.1 | Chronic | Med | 32.1 | 51.9 |  |  | 3.0 |
| Chiappelli | 2015 | Glu | 38 | 7.6 | 1.3 | 36.0 | 7.7 | 1.3 | Chronic | Med | 39.2 | 70.3 |  |  | 3.0 |
| Bustillo | 2010 | Gln | 10 | 4.3 | 1.9 | 10.0 | 3.2 | 1.5 | FEP | Naive | 28.0 | 83.3 |  |  | 4.0 |
| Bernier | 2016 | Glx | 27 | 4.8 | 0.8 | 23.0 | 5.0 | 0.7 | FEP | Med | 24.9 | 62.0 | 52.1 |  | 4.0 |
| Chang | 2007 | Glx | 23 | 4.3 | 1.2 | 22.0 | 3.9 | 0.9 | Chronic | Med | 68.2 |  | 75.5 |  | 4.0 |
| Choe | 1994 | Glx | 23 | 1.1 | 0.3 | 10.0 | 0.9 | 0.2 | Chronic | Naive |  |  |  |  |  |
| Choe | 1996 | Glx | 55 | 0.7 | 0.2 | 20.0 | 0.6 | 0.1 | Chronic | Naive |  | 46.7 |  |  | 1.5 |
| Curcic-Blake | 2017 | Glx | 22 | 10.5 | 1.4 | 15.0 | 10.9 | 1.2 | Chronic | Med | 30.4 | 75.0 | 61.8 |  | 3.0 |
| Curcic-Blake | 2017 | Glx | 45 | 9.8 | 1.1 | 15.0 | 10.9 | 1.2 | Chronic | Med | 29.3 | 73.3 | 61.3 |  | 3.0 |
| Galinska | 2009 | Glx | 30 | 2.1 | 0.4 | 19.0 | 2.3 | 0.6 | FEP | Med | 22.5 | 67.3 | 80.2 |  | 1.5 |
| Galinska | 2018 | Glx | 21 | 0.5 | 0.1 | 20.0 | 0.6 | 0.1 | Chronic | Med | 37.4 | 51.2 | 86.3 |  | 1.5 |
| Ota | 2012 | Glx | 22 | 7.2 | 1.3 | 13.0 | 7.1 | 1.3 | Chronic | Med | 43.9 | 47.1 | 69.2 |  | 1.5 |
| Ota | 2012 | Glx | 20 | 7.3 | 1.0 | 13.0 | 6.5 | 0.9 | Chronic | Med | 42.0 | 51.0 | 56.2 |  | 1.5 |
| Ota | 2015 | Glx | 17 | 6.8 | 1.1 | 22.0 | 7.0 | 1.3 | Chronic | Med | 41.2 | 51.3 | 62.7 | 958.5 | 1.5 |
| Szulc | 2004 | Glx | 31 | 2.0 | 0.6 | 6.5 | 2.2 | 0.7 | FEP | Med | 23.4 |  | 79.7 |  | 1.5 |
| Szulc | 2004 | Glx | 17 | 2.2 | 0.8 | 6.5 | 2.2 | 0.7 | Chronic | Med | 28.9 |  | 88.6 |  | 1.5 |
| Szulc | 2011 | Glx | 40 | 2.2 | 0.4 | 25.0 | 2.1 | 0.4 | Chronic | Med | 31.1 | 67.6 | 94.7 |  | 1.5 |

| *Temporal Lobe* | | | | | | | | | | | | | | | |
| --- | --- | --- | --- | --- | --- | --- | --- | --- | --- | --- | --- | --- | --- | --- | --- |
| Author | Year |  | Patient (n) | Metabolite Mean | SD | HV (n) | Metabolite Mean | SD | Illness Stage | Med | Mean Age | % Male | PANSS Total | CPZ | Tesla |
| Balz | 2018 | Glu | 19 | 9.2 | 1.1 | 21.0 | 8.2 | 1.2 | Chronic | Med | 36.3 | 65.0 |  |  | 3.0 |
| Hjelmervik | 2020 | Glu | 35 | 16.4 | 2.0 | 35.0 | 15.3 | 2.5 | Chronic | Med | 30.0 |  | 62.5 |  | 3.0 |
| Korenic | 2020 | Glu | 19 | 8.2 | 1.3 | 22.0 | 8.5 | 1.3 | Chronic | Med | 29.9 | 63.4 |  |  | 3.0 |
| Bartha | 1999 | Glu | 11 | 7.0 | 2.4 | 11.0 | 7.5 | 1.7 | FEP | Naive | 26.6 | 81.8 |  |  | 1.5 |
| Da Silva | 2011 | Glu | 9 | 7.0 | 1.0 | 16.0 | 6.3 | 0.6 | Chronic | Med | 30.2 | 58.8 | 59.0 |  | 3.0 |
| Gallinat | 2016 | Glu | 29 | 12.1 | 1.5 | 29.0 | 10.4 | 1.5 | Chronic | Med | 29.2 | 65.5 | 72.7 |  | 3.0 |
| Lutkenhoff | 2010 | Glu | 9 | 7.5 | 1.9 | 21.0 | 9.3 | 5.7 | Chronic | Med | 52.2 | 56.7 |  |  | 3.0 |
| Nenadic | 2015 | Glu | 9 | 10.0 | 3.2 | 21.0 | 10.3 | 2.1 | FEP | Naive | 24.1 | 56.7 | 53.2 |  | 3.0 |
| Nenadic | 2015 | Glu | 9 | 10.1 | 2.5 | 21.0 | 9.9 | 2.6 | FEP | Naive | 24.1 | 56.7 | 53.2 |  | 3.0 |
| Onwordi | 2021 | Glu | 18 | 0.9 | 0.0 | 22.0 | 1.0 | 0.0 | Chronic | Med | 39.6 | 90.0 |  |  | 3.0 |
| Shakory | 2018 | Glu | 16 | 10.3 | 1.2 | 31.0 | 10.5 | 1.4 | FEP | Naive | 23.4 | 59.6 |  |  | 3.0 |
| Stan | 2014 | Glu | 18 | 0.8 | 0.1 | 16.0 | 0.9 | 0.1 | Chronic | Med | 38.8 | 67.0 |  |  | 3.0 |
| Singh | 2018 | Glu | 28 | 1.1 | 0.2 | 28.0 | 1.2 | 0.2 | Chronic | Med | 32.7 | 46.4 |  | 488.1 | 3.0 |
| Wijtenburg | 2021 | Glu | 34 | 6.8 | 0.9 | 31.0 | 7.0 | 0.8 | Chronic | Med | 32.4 | 53.8 |  | 336.7 | 7.0 |
| Hjelmervik | 2020 | Gln | 35 | 7.0 | 3.5 | 35.0 | 7.1 | 3.7 | Chronic | Med | 30.0 |  | 62.5 |  | 3.0 |
| Bartha | 1999 | Gln | 11 | 5.3 | 1.5 | 11.0 | 5.0 | 2.4 | FEP | Naive | 26.6 | 81.8 |  |  | 1.5 |
| Da Silva | 2011 | Gln | 9 | 3.9 | 1.7 | 16.0 | 3.0 | 0.8 | Chronic | Med | 30.2 | 58.8 | 59.0 |  | 3.0 |
| Wijtenburg | 2021 | Gln | 29 | 1.8 | 0.5 | 27.0 | 1.7 | 0.6 | Chronic | Med | 32.4 | 53.8 |  | 336.7 | 7.0 |
| Briend | 2020 | Glx | 54 | 13.3 | 3.8 | 41.0 | 12.7 | 3.0 | FEP | Naive | 24.3 | 63.1 |  |  | 3.0 |
| Bustillo | 2020 | Glx | 36 | 17.7 | 3.4 | 29.0 | 21.8 | 1.8 | FEP | Med | 22.9 | 63.1 |  | 98.0 | 3.0 |
| Chang | 2007 | Glx | 23 | 6.4 | 1.9 | 22.0 | 4.9 | 1.3 | Chronic | Med | 68.2 |  | 75.5 |  | 4.0 |
| Da Silva | 2011 | Glx | 9 | 10.9 | 1.7 | 16.0 | 9.3 | 0.9 | Chronic | Med | 30.2 | 58.8 | 59.0 |  | 3.0 |
| Galinska | 2009 | Glx | 30 | 2.5 | 0.6 | 19.0 | 2.5 | 0.7 | FEP | Med | 22.5 | 67.3 | 80.2 |  | 1.5 |
| Galinska | 2018 | Glx | 21 | 0.6 | 0.1 | 20.0 | 0.5 | 0.1 | Chronic | Med | 37.4 | 51.2 | 86.3 |  | 1.5 |
| Hasan | 2014 | Glx | 46 | 2.4 | 0.6 | 24.0 | 2.6 | 0.7 | FEP | Med | 33.6 | 54.3 | 91.9 |  | 1.5 |
| Hjelmervik | 2020 | Glx | 37 | 22.3 | 3.2 | 38.0 | 21.1 | 4.3 | Chronic | Med | 30.0 |  | 62.5 |  | 3.0 |
| Hutcheson | 2012 | Glx | 28 | 0.6 | 0.1 | 28.0 | 0.6 | 0.1 | Chronic | Med | 36.1 | 66.1 | 72.0 |  | 3.0 |
| Kegeles | 2000 | Glx | 10 | 0.9 | 0.4 | 10.0 | 0.8 | 0.4 | Chronic | Med | 28.5 | 100.0 | 59.0 |  | 1.5 |
| Kraguljac | 2012 | Glx | 47 | 0.6 | 0.1 | 44.0 | 0.6 | 0.1 | Chronic | Med | 37.1 | 62.8 | 58.0 |  | 3.0 |
| Kraguljac | 2019 | Glx | 61 | 0.6 | 0.1 | 31.0 | 0.6 | 0.1 | Chronic | Med | 28.2 | 71.5 |  |  | 3.0 |
| Onwordi | 2021 | Glx | 18 | 1.3 | 0.1 | 22.0 | 1.4 | 0.1 | Chronic | Med | 39.6 | 90.0 |  |  | 3.0 |
| Shakory | 2018 | Glx | 16 | 13.4 | 1.1 | 31.0 | 13.6 | 1.6 | FEP | Naive | 23.4 | 59.6 |  |  | 3.0 |
| Singh | 2018 | Glx | 28 | 2.0 | 0.4 | 28.0 | 2.1 | 0.4 | Chronic | Med | 32.7 | 46.4 |  | 488.1 | 3.0 |
| Szulc | 2004 | Glx | 31 | 2.6 | 0.6 | 6.5 | 2.2 | 0.5 | FEP | Med | 23.4 |  | 79.7 |  | 1.5 |
| Szulc | 2004 | Glx | 17 | 2.5 | 0.7 | 6.5 | 2.2 | 0.5 | Chronic | Med | 33.6 |  | 88.6 |  | 1.5 |
| Szulc | 2011 | Glx | 36 | 2.4 | 0.4 | 25.0 | 2.2 | 0.4 | Chronic | Med | 31.1 | 67.6 | 94.7 |  | 1.5 |
| Wood | 2008 | Glx | 12 | 6.4 | 1.5 | 9.5 | 6.4 | 1.6 | FEP | Naive | 20.6 | 70.6 | 74.4 |  | 3.0 |
| Wood | 2008 | Glx | 13 | 6.9 | 1.0 | 9.5 | 6.4 | 1.6 | FEP | Med | 20.6 | 63.2 | 90.3 |  | 3.0 |
| Wood | 2010 | Glx | 6 | 8.0 | 1.3 | 14.5 | 6.6 | 0.7 | FEP | Naive | 20.2 |  |  |  | 3.0 |

| *Thalamus* |  |  |  |  |  |  |  |  |  |  |  |  |  |  |  |
| --- | --- | --- | --- | --- | --- | --- | --- | --- | --- | --- | --- | --- | --- | --- | --- |
| Author | Year |  | Patient (n) | Metabolite Mean | SD | HV (n) | Metabolite Mean | SD | Illness Stage | Med | Mean Age | % Male | PANSS Total | CPZ | Tesla |
| Bojesen | 2021 | Glu | 48 | 7.0 | 0.9 | 46.0 | 6.8 | 0.8 | FEP | Naive | 22.4 | 43.0 | 75.9 |  | 3.0 |
| Bustillo | 2010 | Glu | 12 | 12.3 | 3.4 | 10.0 | 11.5 | 4.3 | FEP | Naive | 28.0 | 83.3 |  |  | 4.0 |
| Egerton | 2018 | Glu | 24 | 1.0 | 0.1 | 36.0 | 1.1 | 0.1 | FEP | Med | 27.9 | 76.7 | 65.4 |  | 3.0 |
| Egerton | 2018 | Glu | 27 | 1.1 | 0.2 | 14.0 | 1.2 | 0.2 | FEP | Med | 22.9 | 58.5 | 83.2 |  | 3.0 |
| Egerton | 2018 | Glu | 11 | 1.2 | 0.2 | 8.0 | 1.0 | 0.2 | FEP | Med | 25.3 | 94.7 | 70.4 |  | 3.0 |
| Legind | 2019 | Glu | 23 | 7.5 | 0.8 | 52.0 | 7.1 | 0.8 | Chronic | Med | 39.6 | 60.0 | 62.9 |  | 3.0 |
| Legind | 2019 | Glu | 22 | 7.4 | 1.1 | 36.0 | 7.3 | 0.9 | Chronic | Med | 42.2 | 56.9 | 60.6 |  | 3.0 |
| Roalf | 2017 | Glu | 5 | 4.7 | 0.4 | 17.0 | 5.4 | 0.4 | FEP | Med | 20.7 | 54.5 |  |  | 7.0 |
| Smesny | 2021 | Glu | 29 | 6.8 | 0.9 | 26.0 | 7.4 | 0.6 | FEP | Naive | 25.5 | 47.6 |  |  | 3.0 |
| Taylor | 2017 | Glu | 16 | 7.4 | 1.0 | 18.0 | 7.4 | 0.6 | Chronic | Med | 23.3 | 70.6 |  | 358.0 | 7.0 |
| Theberge | 2002 | Glu | 19 | 14.1 | 3.3 | 19.0 | 14.0 | 2.3 | FEP | Naive | 26.0 | 66.7 |  |  | 4.0 |
| Theberge | 2003 | Glu | 19 | 13.1 | 1.9 | 19.0 | 13.1 | 1.7 | Chronic | Med | 35.0 | 95.2 |  |  | 4.0 |
| Wang | 2019 | Glu | 66 | 6.2 | 0.6 | 74.0 | 6.4 | 0.5 | FEP | Med | 22.8 | 58.3 |  | 188.0 | 7.0 |
| Wijtenburg | 2021 | Glu | 40 | 6.9 | 0.9 | 38.0 | 6.9 | 0.7 | Chronic | Med | 32.4 | 53.8 |  | 336.7 | 7.0 |
| Batalla | 2015 | Glx | 10 | 4.8 | 1.3 | 6.0 | 5.4 | 1.7 | FEP | Naive | 23.1 | 50.0 | 117.4 |  | 3.0 |
| Bojesen | 2021 | Glx | 48 | 9.9 | 1.7 | 47.0 | 9.8 | 1.4 | FEP | Naive | 22.4 | 43.0 | 75.9 |  | 3.0 |
| Egerton | 2018 | Glx | 26 | 1.3 | 0.3 | 36.0 | 1.4 | 0.3 | FEP | Med | 27.9 | 74.2 | 65.4 |  | 3.0 |
| Egerton | 2018 | Glx | 27 | 1.6 | 0.3 | 14.0 | 1.5 | 0.2 | FEP | Med | 22.9 | 58.5 | 83.2 |  | 3.0 |
| Egerton | 2018 | Glx | 12 | 1.6 | 0.3 | 7.0 | 1.4 | 0.4 | FEP | Med | 25.3 | 94.7 | 70.4 |  | 3.0 |
| Galinska | 2009 | Glx | 30 | 1.9 | 0.4 | 19.0 | 1.9 | 0.2 | FEP | Med | 22.5 | 67.3 | 80.2 |  | 1.5 |
| Legind | 2019 | Glx | 24 | 10.6 | 1.1 | 52.0 | 10.2 | 1.3 | Chronic | Med | 39.6 | 59.2 | 62.9 |  | 3.0 |
| Legind | 2019 | Glx | 22 | 10.8 | 1.5 | 36.0 | 10.3 | 1.0 | Chronic | Med | 42.2 | 56.9 | 60.6 |  | 3.0 |
| Smesny | 2021 | Glx | 29 | 10.0 | 1.6 | 26.0 | 10.5 | 1.1 | FEP | Naive | 25.5 | 47.6 |  |  | 3.0 |
| Szulc | 2004 | Glx | 31 | 2.0 | 0.6 | 6.5 | 1.8 | 0.3 | FEP | Med | 23.4 |  | 79.7 |  | 1.5 |
| Szulc | 2004 | Glx | 17 | 1.8 | 0.4 | 6.5 | 1.8 | 0.3 | Chronic | Med | 28.9 |  | 88.6 |  | 1.5 |
| Szulc | 2011 | Glx | 42 | 1.8 | 0.2 | 26.0 | 1.8 | 0.2 | Chronic | Med | 31.1 | 67.6 | 94.7 |  | 1.5 |
| Wang | 2019 | Glx | 67 | 7.8 | 0.9 | 74.0 | 7.9 | 0.7 | FEP | Med | 22.8 | 58.3 |  | 188.0 | 7.0 |
| Bustillo | 2010 | Gln | 12 | 6.4 | 3.3 | 10.0 | 6.7 | 2.4 | FEP | Naive | 28.0 | 83.3 |  |  | 4.0 |
| Taylor | 2017 | Gln | 16 | 1.9 | 0.6 | 18.0 | 1.3 | 0.6 | Chronic | Med | 23.3 | 70.6 |  | 358.0 | 7.0 |
| Theberge | 2002 | Gln | 19 | 8.6 | 2.1 | 19.0 | 7.3 | 1.6 | FEP | Naive | 26.0 | 66.7 |  |  | 4.0 |
| Theberge | 2003 | Gln | 19 | 8.8 | 2.0 | 19.0 | 6.6 | 2.6 | Chronic | Med | 35.0 | 95.2 |  |  | 4.0 |
| Wang | 2019 | Gln | 52 | 1.6 | 0.4 | 68.0 | 1.5 | 0.3 | FEP | Med | 22.8 | 58.3 |  | 188.0 | 7.0 |
| Wijtenburg | 2021 | Gln | 38 | 1.9 | 0.6 | 38.0 | 1.6 | 0.3 | Chronic | Med | 32.4 | 53.8 |  | 336.7 | 7.0 |

| *Basal Ganglia* | | | | | | | | | | | | | | | |
| --- | --- | --- | --- | --- | --- | --- | --- | --- | --- | --- | --- | --- | --- | --- | --- |
| Author | Year |  | Patient (n) | Metabolite Mean | SD | HV (n) | Metabolite Mean | SD | Illness Stage | Med | Mean Age | % Male | PANSS Total | CPZ | Tesla |
| Godlewska | 2021 | Glu | 16 | 9.6 | 0.2 | 18.0 | 9.7 | 0.2 | FEP | Med | 26.4 | 100.0 | 58.0 |  | 7.0 |
| Goldstein | 2015 | Glu | 12 | 1.1 | 0.1 | 4.3 | 1.2 | 0.1 | Chronic | Med | 32.4 | 80.3 | 59.9 | 426.5 | 3.0 |
| Goldstein | 2015 | Glu | 8 | 1.2 | 0.2 | 4.3 | 1.2 | 0.1 | Chronic | Med | 33.9 | 76.6 | 59.7 | 446.0 | 3.0 |
| Goldstein | 2015 | Glu | 9 | 1.1 | 0.2 | 4.3 | 1.2 | 0.1 | Chronic | Med | 34.5 | 81.6 | 62.4 | 855.4 | 3.0 |
| Iwata | 2019 | Glu | 24 | 11.4 | 1.7 | 8.7 | 11.5 | 2.2 | Chronic | Med | 43.0 | 77.0 | 82.8 | 643.2 | 3.0 |
| Iwata | 2019 | Glu | 25 | 11.9 | 1.7 | 8.7 | 11.5 | 2.2 | Chronic | Med | 40.6 | 71.8 | 56.1 | 527.1 | 3.0 |
| Iwata | 2019 | Glu | 21 | 12.1 | 1.8 | 8.7 | 11.5 | 2.2 | Chronic | Med | 43.5 | 74.7 | 57.2 | 443.1 | 3.0 |
| Plitman | 2016 | Glu | 64 | 13.1 | 1.3 | 63.0 | 12.4 | 0.9 | FEP | Naive | 23.9 | 62.5 | 97.2 |  | 3.0 |
| Plitman | 2018 | Glu | 12 | 11.3 | 1.2 | 11.0 | 11.7 | 1.2 | Chronic | Med | 42.9 | 65.2 |  | 471.2 | 3.0 |
| Thakkar | 2017 | Glu | 10 | 9.4 | 1.5 | 12.0 | 9.5 | 1.6 | Chronic | Med | 35.1 | 68.9 | 49.1 | 281.4 | 7.0 |
| Thakkar | 2017 | Glu | 10 | 7.7 | 1.3 | 12.0 | 7.5 | 1.5 | Chronic | Med | 35.1 | 68.9 | 49.1 | 281.4 | 7.0 |
| Tayoshi | 2009 | Glu | 30 | 4.8 | 0.8 | 25.0 | 4.8 | 0.8 | Chronic | Med | 34.4 | 49.1 | 55.2 |  | 3.0 |
| Godlewska | 2021 | Gln | 16 | 3.2 | 0.2 | 18.0 | 3.0 | 0.1 | FEP | Med | 26.4 | 100.0 | 58.0 |  | 7.0 |
| Tayoshi | 2009 | Gln | 30 | 2.7 | 0.7 | 25.0 | 2.7 | 0.6 | Chronic | Med | 34.4 | 49.1 | 55.2 |  | 3.0 |
| Thakkar | 2017 | Gln | 10 | 3.2 | 1.2 | 12.0 | 3.0 | 1.1 | Chronic | Med | 35.1 | 68.9 | 49.1 | 281.4 | 7.0 |
| Thakkar | 2017 | Gln | 10 | 2.9 | 1.0 | 12.0 | 3.5 | 1.7 | Chronic | Med | 35.1 | 68.9 | 49.1 | 281.4 | 7.0 |
| Block | 2000 | Glx | 25 | 1.4 | 0.4 | 19.0 | 1.2 | 0.4 | Chronic | Med | 37.9 | 56.8 |  |  | 1.5 |
| de la Fuente-Sandoval | 2018 | Glx | 28 | 1.6 | 0.3 | 18.0 | 1.4 | 0.2 | FEP | Naive | 23.0 | 63.0 | 104.4 |  | 3.0 |
| Goldstein | 2015 | Glx | 12 | 1.5 | 0.1 | 4.3 | 1.5 | 0.1 | Chronic | Med | 32.4 | 80.3 | 59.9 | 426.5 | 3.0 |
| Goldstein | 2015 | Glx | 8 | 1.8 | 0.1 | 4.3 | 1.5 | 0.1 | Chronic | Med | 33.9 | 76.6 | 59.7 | 446.0 | 3.0 |
| Goldstein | 2015 | Glx | 9 | 1.4 | 0.1 | 4.3 | 1.5 | 0.1 | Chronic | Med | 34.5 | 81.6 | 62.4 | 855.4 | 3.0 |
| Goto | 2012 | Glx | 18 | 0.7 | 0.2 | 18.0 | 0.6 | 0.2 | FEP | Med | 30.0 | 50.0 | 68.1 |  | 3.0 |
| Iwata | 2019 | Glx | 25 | 16.3 | 2.8 | 8.7 | 15.8 | 2.2 | Chronic | Med | 43.0 | 77.0 | 82.8 | 643.2 | 3.0 |
| Iwata | 2019 | Glx | 25 | 16.2 | 1.9 | 8.7 | 15.8 | 2.2 | Chronic | Med | 40.6 | 71.8 | 56.1 | 527.1 | 3.0 |
| Iwata | 2019 | Glx | 21 | 16.9 | 2.3 | 8.7 | 15.8 | 2.2 | Chronic | Med | 43.5 | 74.7 | 57.2 | 443.1 | 3.0 |
| Plitman | 2016 | Glx | 64 | 16.6 | 1.7 | 63.0 | 16.2 | 1.5 | FEP | Naive | 23.9 | 62.5 | 97.2 |  | 3.0 |
| Plitman | 2018 | Glx | 12 | 15.3 | 1.9 | 11.0 | 15.8 | 1.8 | Chronic | Med | 42.9 | 65.2 |  | 471.2 | 3.0 |
| Reid | 2013 | Glx | 35 | 0.6 | 0.2 | 22.0 | 0.6 | 0.2 | Chronic | Med | 37.9 | 68.4 |  |  | 3.0 |
| Sivaraman | 2018 | Glx | 14 | 0.2 | 0.0 | 18.0 | 0.2 | 0.1 | FEP | Naive | 23.0 | 65.6 |  |  | 3.0 |
| Tarumi | 2020 | Glx | 23 | 14.1 | 2.1 | 11.5 | 13.6 | 2.8 | Chronic | Med | 43.8 | 44.0 | 111.2 | 958.5 | 3.0 |
| Tarumi | 2020 | Glx | 27 | 13.7 | 2.7 | 11.5 | 13.6 | 2.8 | Chronic | Med | 43.0 | 45.5 | 49.8 | 412.4 | 3.0 |
| Thakkar | 2017 | Glx | 10 | 12.6 | 2.5 | 12.0 | 12.5 | 2.5 | Chronic | Med | 35.1 | 68.9 | 49.1 | 281.4 | 7.0 |
| Thakkar | 2017 | Glx | 10 | 10.6 | 1.7 | 12.0 | 11.0 | 2.2 | Chronic | Med | 35.1 | 68.9 | 49.1 | 281.4 | 7.0 |
| Yamasue | 2003 | Glx | 16 | 13.9 | 3.0 | 15.0 | 12.6 | 2.0 | Chronic | Med | 29.6 | 61.3 | 68.5 |  | 1.5 |

## eTable 2: Quality assessment of studies included in meta-analysis.

The Newcastle Ottawa Scale considers study quality based on three domains: 1) Quality of subject ‘selection’ (cases and controls, maximum 4 points); 2) Quality of ‘comparability’ of cases and controls (defined as matching for age, maximum 1 point); 3) Quality of ‘exposure’ (defined as use of structured interview to define patients as experiencing a first episode of psychosis or use of DSM/ICD diagnoses of schizophrenia, schizoaffective disorder, schizophreniform disorder, schizophrenia spectrum or psychotic disorder not otherwise specified, and use of structured interview to define absence of mental illness in healthy controls, maximum 2 points). Item ‘non-response rate’ for Quality of exposure in the scale was not applicable. A maximum of 7 points was therefore considered.

| Study | Selection | | | | Comparability | Exposure | | Total Score | Quality Rating |
| --- | --- | --- | --- | --- | --- | --- | --- | --- | --- |
|  | 1 | 2 | 3 | 4 | Age | 1 | 2 |  |  |
|  |  |  |  |  |  |  |  |  |  |
| Onwordi 2021 | 1 | 1 | 0 | 1 | 1 | 1 | 1 | 6 | Good |
| Godlewska 2021 | 1 | 1 | 0 | 1 | 1 | 1 | 0 | 5 | Fair |
| Bustillo 2021 | 1 | 1 | 1 | 1 | 1 | 1 | 1 | 7 | Good |
| Wijtenburg 2021 | 1 | 0 | 0 | 1 | 1 | 1 | 1 | 5 | Fair |
| Bryant 2021 | 1 | 1 | 0 | 1 | 1 | 1 | 1 | 6 | Good |
| Falkenberg 2014 | 0 | 0 | 0 | 1 | 1 | 0 | 0 | 2 | Poor |
| Natsubori 2014 | 1 | 1 | 1 | 1 | 1 | 1 | 1 | 7 | Good |
| Goto 2012 | 1 | 0 | 0 | 1 | 1 | 0 | 0 | 3 | Poor |
| Smesny 2021 | 1 | 1 | 0 | 1 | 1 | 1 | 0 | 5 | Fair |
| Coughlin 2020 | 0 | 0 | 1 | 1 | 1 | 1 | 0 | 4 | Fair |
| Bojesen 2021 | 1 | 1 | 0 | 0 | 1 | 1 | 0 | 4 | Fair |
| Bryll 2020 | 1 | 1 | 0 | 1 | 1 | 1 | 0 | 5 | Fair |
| Bustillo 2020 | 1 | 1 | 1 | 1 | 1 | 1 | 0 | 6 | Fair |
| Briend 2020 | 1 | 1 | 0 | 1 | 1 | 1 | 0 | 5 | Fair |
| Corcoran 2020 | 1 | 1 | 0 | 1 | 1 | 1 | 0 | 5 | Fair |
| Cen 2020 | 1 | 1 | 1 | 1 | 1 | 1 | 1 | 7 | Good |
| Ragland 2020 | 0 | 0 | 0 | 0 | 1 | 0 | 0 | 1 | Poor |
| Tarumi 2020 | 1 | 1 | 0 | 1 | 1 | 1 | 1 | 6 | Good |
| Hjelmervik 2020 | 1 | 1 | 0 | 0 | 1 | 1 | 0 | 4 | Fair |
| Korenic 2020 | 1 | 1 | 1 | 0 | 1 | 1 | 1 | 6 | Good |
| Kaminski 2020 | 1 | 1 | 0 | 1 | 1 | 1 | 1 | 6 | Good |
| Bartolomeo 2019 | 1 | 1 | 1 | 0 | 0 | 1 | 1 | 5 | Poor |
| Pillinger 2019 | 1 | 1 | 1 | 1 | 1 | 1 | 0 | 6 | Fair |
| Xiang 2019 | 1 | 1 | 1 | 1 | 1 | 1 | 1 | 7 | Good |
| Kraguljac 2019 | 1 | 1 | 0 | 1 | 1 | 1 | 0 | 5 | Fair |
| Wang 2019 | 1 | 1 | 0 | 0 | 1 | 1 | 0 | 4 | Fair |
| Galinska 2018 | 1 | 1 | 0 | 0 | 1 | 1 | 0 | 4 | Fair |
| Iwata 2019 | 1 | 1 | 0 | 1 | 1 | 1 | 1 | 6 | Fair |
| Legind 2019 | 0 | 0 | 0 | 0 | 1 | 0 | 0 | 1 | Poor |
| Sivaraman 2018 | 1 | 1 | 1 | 1 | 1 | 1 | 0 | 6 | Fair |
| Jauhar 2018 | 1 | 1 | 1 | 1 | 1 | 1 | 1 | 7 | Good |
| Kumar 2020 | 1 | 1 | 1 | 1 | 1 | 1 | 0 | 6 | Fair |
| Egerton 2018 | 1 | 1 | 0 | 1 | 0 | 1 | 0 | 4 | Poor |
| Balz 2018 | 1 | 1 | 0 | 0 | 1 | 1 | 1 | 5 | Fair |
| Posporelis 2018 | 1 | 1 | 1 | 0 | 1 | 1 | 1 | 6 | Good |
| Roalf 2017 | 1 | 0 | 0 | 1 | 1 | 0 | 1 | 4 | Fair |
| Batalla 2015 | 1 | 1 | 0 | 1 | 1 | 1 | 1 | 6 | Good |
| Coughlin 2015 | 1 | 1 | 0 | 1 | 1 | 1 | 0 | 5 | Fair |
| Goldstein 2015 | 1 | 1 | 1 | 1 | 1 | 1 | 0 | 6 | Fair |
| Marsman 2014 | 1 | 0 | 0 | 1 | 1 | 0 | 0 | 3 | Poor |
| Choe 1994 | 1 | 0 | 0 | 1 | 0 | 0 | 0 | 2 | Poor |
| Stanley 1996 | 1 | 0 | 0 | 1 | 1 | 1 | 1 | 5 | Fair |
| Choe 1996 | 1 | 0 | 0 | 1 | 0 | 0 | 0 | 2 | Poor |
| Bartha 1997 | 1 | 0 | 0 | 1 | 1 | 0 | 1 | 4 | Fair |
| Thomas 1998 | 1 | 0 | 0 | 0 | 1 | 0 | 0 | 2 | Poor |
| Bartha 1999 | 1 | 0 | 0 | 1 | 1 | 0 | 1 | 4 | Fair |
| Kegeles 2000 | 1 | 0 | 0 | 1 | 1 | 0 | 1 | 4 | Fair |
| Block 2000 | 1 | 0 | 0 | 0 | 1 | 0 | 0 | 2 | Poor |
| Theberge 2002 | 1 | 0 | 0 | 1 | 0 | 0 | 1 | 3 | Poor |
| Yamasue 2003 | 1 | 1 | 0 | 1 | 1 | 1 | 0 | 5 | Fair |
| Theberge 2003 | 1 | 0 | 0 | 1 | 0 | 1 | 1 | 4 | Poor |
| Ohrmann 2005 | 1 | 1 | 0 | 1 | 1 | 1 | 0 | 5 | Fair |
| Terpstra 2005 | 0 | 0 | 0 | 0 | 0 | 0 | 1 | 1 | Poor |
| Ohrmann 2007 | 1 | 1 | 0 | 0 | 1 | 1 | 0 | 4 | Fair |
| Stanley 2007 | 1 | 1 | 0 | 1 | 1 | 1 | 1 | 6 | Good |
| Wood 2007 | 1 | 1 | 0 | 1 | 1 | 1 | 1 | 6 | Good |
| Wood 2008 | 0 | 0 | 0 | 1 | 1 | 0 | 1 | 3 | Fair |
| Lutkenhoff 2010 | 1 | 1 | 0 | 0 | 1 | 1 | 0 | 4 | Fair |
| Ohrmann 2008 | 1 | 1 | 0 | 1 | 1 | 1 | 1 | 6 | Good |
| Rowland 2009 | 1 | 0 | 0 | 1 | 1 | 0 | 1 | 4 | Fair |
| Tayoshi 2009 | 1 | 1 | 0 | 1 | 1 | 1 | 1 | 6 | Good |
| Galinska 2009 | 1 | 1 | 0 | 0 | 1 | 1 | 0 | 4 | Fair |
| Tunc-skarka 2009 | 1 | 1 | 0 | 0 | 0 | 1 | 0 | 3 | Poor |
| Shirayama 2010 | 1 | 1 | 0 | 1 | 1 | 1 | 0 | 5 | Fair |
| Bustillo 2010 | 1 | 1 | 0 | 1 | 1 | 1 | 1 | 6 | Good |
| Wood 2010 | 1 | 1 | 0 | 1 | 1 | 1 | 1 | 6 | Good |
| Ongur 2010 | 1 | 1 | 0 | 0 | 0 | 1 | 0 | 3 | Poor |
| Szulc 2011 | 1 | 1 | 0 | 1 | 1 | 1 | 1 | 6 | Good |
| Da silva 2011 | 1 | 0 | 0 | 0 | 1 | 0 | 0 | 2 | Poor |
| Jessen 2013 | 1 | 1 | 0 | 1 | 1 | 1 | 0 | 5 | Fair |
| Hasan 2014 | 1 | 0 | 0 | 1 | 1 | 0 | 0 | 3 | Poor |
| Kegeles 2012 | 1 | 0 | 0 | 1 | 1 | 0 | 1 | 4 | Fair |
| Ota 2012 | 1 | 1 | 0 | 1 | 1 | 1 | 1 | 6 | Good |
| Tibbo 2013 | 1 | 1 | 0 | 0 | 1 | 1 | 1 | 5 | Good |
| Kraguljac 2012 | 1 | 1 | 0 | 0 | 1 | 1 | 0 | 4 | Fair |
| Hutcheson 2012 | 1 | 1 | 0 | 0 | 1 | 1 | 0 | 4 | Fair |
| Rowland 2013 | 1 | 0 | 0 | 1 | 1 | 0 | 1 | 4 | Fair |
| Demjaha 2014 | 1 | 0 | 0 | 1 | 1 | 1 | 0 | 4 | Fair |
| Bustillo 2014 | 1 | 1 | 0 | 1 | 1 | 1 | 1 | 6 | Good |
| Chiu 2017 | 1 | 0 | 0 | 0 | 1 | 0 | 1 | 3 | Fair |
| Huang 2017 | 1 | 1 | 0 | 0 | 1 | 1 | 0 | 4 | Fair |
| Curcic-Blake 2017 | 1 | 0 | 0 | 0 | 0 | 0 | 0 | 1 | Poor |
| Taylor 2017 | 1 | 0 | 0 | 1 | 1 | 0 | 1 | 4 | Fair |
| Wang 2016 | 1 | 1 | 0 | 1 | 1 | 1 | 1 | 6 | Good |
| Rowland 2016 | 1 | 1 | 0 | 1 | 1 | 1 | 1 | 6 | Good |
| Wijtenburg 2017 | 1 | 0 | 0 | 1 | 1 | 0 | 1 | 4 | Fair |
| Thakkar 2017 | 1 | 1 | 0 | 0 | 1 | 1 | 0 | 4 | Fair |
| Bernier 2016 | 1 | 0 | 0 | 1 | 0 | 0 | 0 | 2 | Poor |
| Xin 2016 | 1 | 1 | 0 | 0 | 1 | 1 | 0 | 4 | Fair |
| Brandt 2016 | 1 | 1 | 0 | 1 | 1 | 1 | 1 | 6 | Good |
| Liemburg 2016 | 1 | 1 | 0 | 0 | 0 | 1 | 0 | 3 | Poor |
| Rowland 2016 | 1 | 0 | 0 | 1 | 1 | 0 | 1 | 4 | Fair |
| Gallinat 2016 | 1 | 1 | 0 | 1 | 0 | 1 | 1 | 5 | Poor |
| Plitman 2016 | 1 | 1 | 0 | 1 | 1 | 1 | 1 | 6 | Good |
| Smesny 2015 | 1 | 0 | 0 | 1 | 1 | 0 | 0 | 3 | Poor |
| Nenadic 2015 | 1 | 0 | 0 | 1 | 1 | 0 | 0 | 3 | Poor |
| Ota 2015 | 1 | 1 | 0 | 0 | 1 | 1 | 1 | 5 | Fair |
| Chiappelli 2015 | 1 | 1 | 0 | 0 | 1 | 1 | 1 | 5 | Fair |
| Plitman 2018 | 1 | 0 | 0 | 1 | 1 | 0 | 1 | 4 | Fair |
| Reid 2018 | 1 | 1 | 0 | 0 | 1 | 1 | 0 | 4 | Fair |
| Chen 2017 | 1 | 1 | 0 | 0 | 1 | 1 | 1 | 5 | Fair |
| Singh 2018 | 1 | 1 | 0 | 0 | 1 | 1 | 0 | 4 | Fair |
| De la Fuente-Sandoval 2018 | 1 | 1 | 0 | 1 | 1 | 1 | 1 | 6 | Good |
| Kim 2018 | 1 | 0 | 0 | 1 | 1 | 0 | 1 | 4 | Fair |
| Borgan 2019 | 1 | 1 | 0 | 1 | 1 | 1 | 1 | 6 | Good |
| Dempster 2020 | 1 | 1 | 0 | 0 | 1 | 1 | 0 | 4 | Fair |
| Ongur 2008 | 1 | 1 | 0 | 0 | 0 | 1 | 1 | 4 | Poor |
| Li 2020 | 1 | 1 | 0 | 0 | 1 | 1 | 0 | 4 | Fair |
| Lesh 2021 | 1 | 0 | 0 | 0 | 1 | 0 | 1 | 3 | Fair |
| Stan 2014 | 1 | 0 | 0 | 1 | 1 | 0 | 1 | 4 | Fair |
| Chang 2007 | 1 | 1 | 0 | 1 | 0 | 1 | 1 | 5 | Poor |
| Szulc 2004 | 1 | 1 | 0 | 0 | 1 | 1 | 0 | 4 | Fair |
| Yang 2015 | 1 | 1 | 1 | 1 | 1 | 1 | 1 | 7 | Good |
| Reid 2013 | 1 | 0 | 0 | 0 | 1 | 1 | 0 | 3 | Fair |
| Shakory 2018 | 1 | 0 | 0 | 1 | 1 | 0 | 0 | 3 | Poor |
| Birur 2020 | 1 | 1 | 0 | 1 | 1 | 1 | 0 | 5 | Fair |
| Cai 2022 | 1 | 1 | 1 | 1 | 1 | 1 | 1 | 7 | Good |
| Huang 2022 | 1 | 1 | 1 | 1 | 1 | 1 | 0 | 6 | Good |
| Jeon 2021 | 1 | 1 | 0 | 1 | 1 | 1 | 0 | 5 | Good |
| Leptourgos 2022 | 1 | 1 | 0 | 1 | 1 | 1 | 1 | 6 | Good |
| Smucny 2022 | 1 | 1 | 0 | 1 | 1 | 1 | 0 | 5 | Good |
| Wang 2022 | 1 | 1 | 1 | 1 | 1 | 1 | 1 | 7 | Good |
| Matrone 2022 | 1 | 1 | 0 | 1 | 1 | 1 | 1 | 6 | Good |

Thresholds used for converting the NOS rating to Agency for Healthcare Research and Quality - AHRQ - standards (good, fair, and poor):

Good quality: 3 or 4 stars in Selection domain AND 1 star in Comparability domain AND 1 or 2 stars in Exposure domain

Fair quality: 2 stars in Selection domain AND 1 star in Comparability domain AND 1 or 2 stars in Exposure domain

Poor quality: 0 or 1 star in Selection domain OR 0 stars in Comparability domain OR 0 or 1 stars in Exposure domain

## eFigure 1: PRISMA diagram

**Identification of studies via databases and registers**

Records removed *before screening*:

Duplicate records removed (n = 0)

Records marked as ineligible by automation tools (n = 0)

Records removed for other reasons (n = 0)

Records identified from*:

Databases (n = 2527)

Registers (n = 0)

**Identification**

Records screened

(n = 2527)

Records excluded**

(n = 2142)

Reports sought for retrieval

(n = 385)

Reports not retrieved

(n = 0)

**Screening**

Reports excluded:

- Reviews (n = 70)

- Overlapping sample (n = 66)

- High risk cohort (n = 32)

- Not schizophrenia (n = 23)

- No healthy volunteers (n = 22)

- Glutamate not measured (n = 19)

- Brain region not reported by >3 studies (n = 10)

- Measures not reported (n = 8)

- Other (MRSI/fMRS/) (n=12)

Reports assessed for eligibility

(n = 385)

Studies included in review

(n = 123)

**Included**

eFigure 1 PRISMA 2020 flow diagram for new systematic reviews which included searches of databases and registers only

*From:*  Page MJ, McKenzie JE, Bossuyt PM, Boutron I, Hoffmann TC, Mulrow CD, et al. The PRISMA 2020 statement: an updated guideline for reporting systematic reviews. BMJ 2021;372:n71. doi: 10.1136/bmj.n71

For more information, visit: <http://www.prisma-statement.org/>

## eFigure 2: Correlation between mean glutamatergic metabolite level and standard deviation


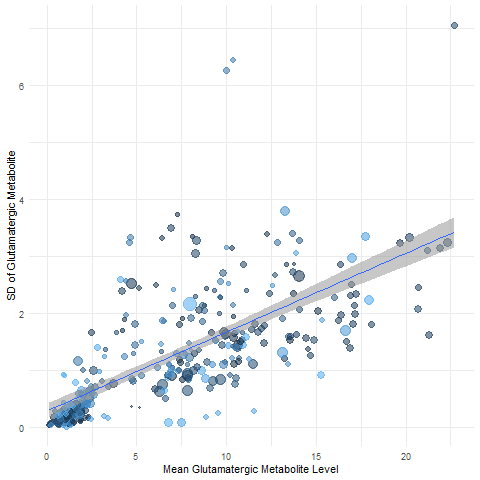


eFigure 2. A significant correlation exists between mean glutamatergic metabolite level and the standard deviation for each study (weighted rp=.70, p<0.001). The shaded area represents the 95% confidence interval, and the size of the dots is proportional to study size.

## eFigure 3: Forest plot of variation ratio (VR)


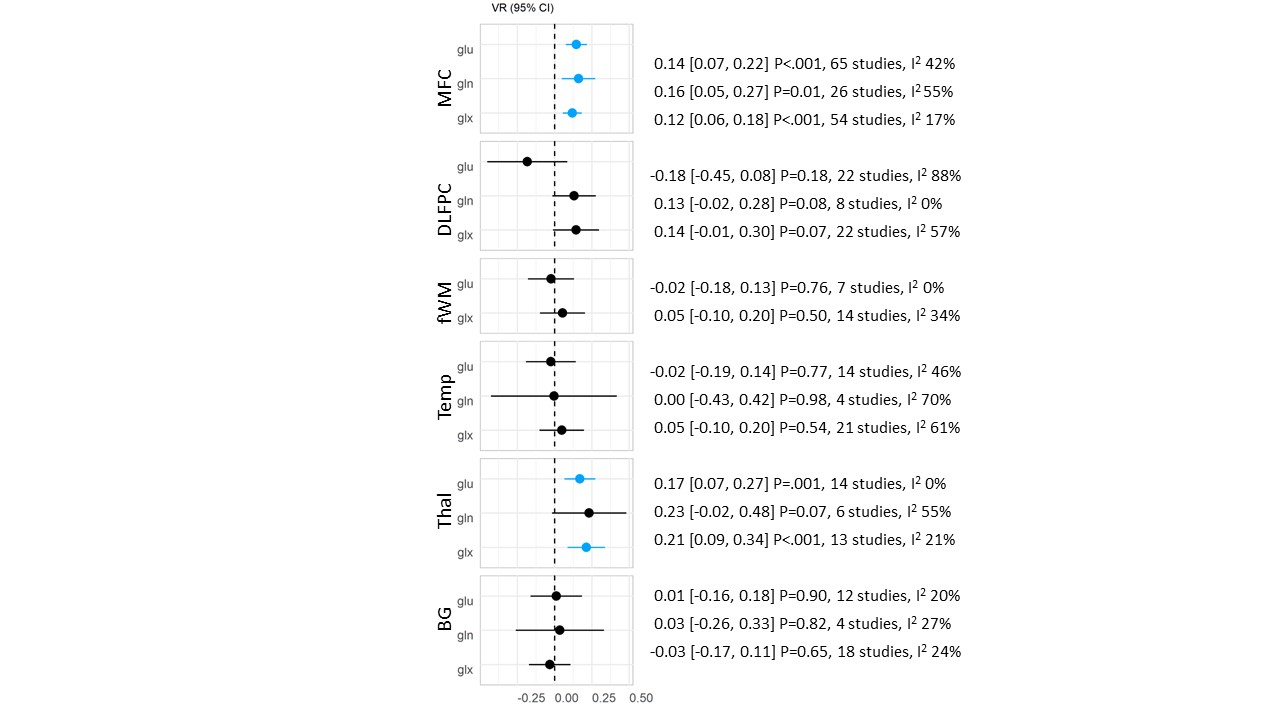


eFigure 3. Forest plot showing the summary effect sizes for the variation ratio (VR) of glutamate measures in schizophrenia patients compared to healthy volunteers (HV). Significant results are shown in blue. Variability was significantly higher in patients relative to HV in the medial frontal cortex (MFC; all glutamatergic metabolites) and Thalamus (Thal; Glu and Gln). There were no significant differences in glutamatergic metabolite variability in the frontal white matter (fWM), temporal lobe (Temp) and basal ganglia (BG) in patients compared with HV, and a trend for higher glutamine and Glx was seen in the dorsolateral prefrontal cortex (DLPFC). Please note, CVR is a more appropriate measure than VR, as mean glutamatergic metabolite levels correlate with standard deviation. Glu = glutamate, Gln = glutamine. CVR, 95% confidence intervals, P value and I^2^ presented.

## eFigure 4: Meta-regressions for glutamate standardised mean differences

## *Age*


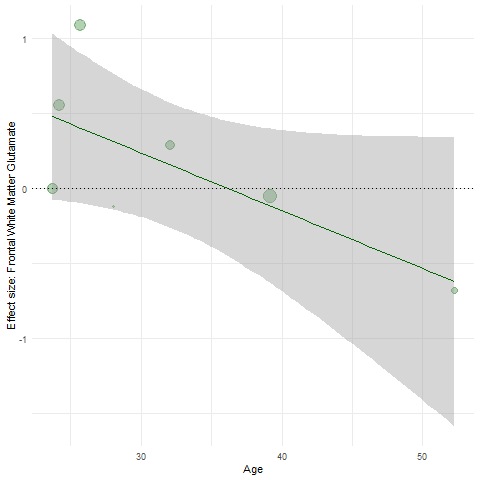

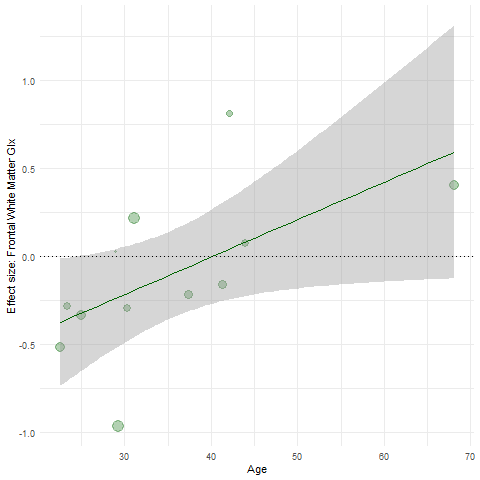

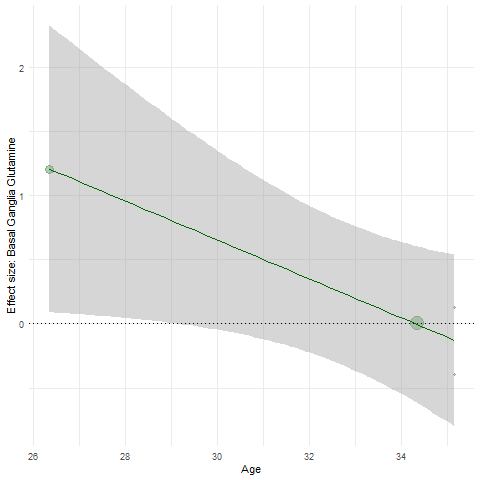


eFigure 4: Meta-regressions of Hedges’ g effect sizes and age. Higher glutamate and glutamine levels in patients were associated with studies in younger participants in the frontal white matter and basal ganglia respectively, whereas elevated Glx in the frontal white matter was associated with older participants. Bubble size represents total sample of patients and HV.

# References

1. Yamasue H, Fukui T, Fukuda R, Kasai K, Iwanami A, Kato N, et al. Drug-induced parkinsonism in relation to choline-containing compounds measured by 1H-MR spectroscopy in putamen of chronically medicated patients with schizophrenia. Int J Neuropsychopharmacol [Internet]. 2003 Dec;6(4):353–60. Available from: http://www.ncbi.nlm.nih.gov/pubmed/14604450

2. Hasan A, Wobrock T, Falkai P, Schneider-Axmann T, Guse B, Backens M, et al. Hippocampal integrity and neurocognition in first-episode schizophrenia: a multidimensional study. World J Biol Psychiatry [Internet]. 2014 Apr [cited 2014 Nov 10];15(3):188–99. Available from: http://www.ncbi.nlm.nih.gov/pubmed/22047183

3. Plitman E, de la Fuente-Sandoval C, Reyes-Madrigal F, Chavez S, Gómez-Cruz G, León-Ortiz P, et al. Elevated Myo-Inositol, Choline, and Glutamate Levels in the Associative Striatum of Antipsychotic-Naive Patients With First-Episode Psychosis: A Proton Magnetic Resonance Spectroscopy Study With Implications for Glial Dysfunction. Schizophr Bull [Internet]. 2016 Mar [cited 2017 Aug 9];42(2):415–24. Available from: https://academic.oup.com/schizophreniabulletin/article-lookup/doi/10.1093/schbul/sbv118

4. Bustillo JR, Chen H, Jones T, Lemke N, Abbott C, Qualls C, et al. Increased glutamine in patients undergoing long-term treatment for schizophrenia: a proton magnetic resonance spectroscopy study at 3 T. JAMA psychiatry [Internet]. 2014 Mar [cited 2014 Nov 10];71(3):265–72. Available from: http://www.ncbi.nlm.nih.gov/pubmed/24402128

5. Block W, Bayer TA, Tepest R, Tra F, Rietschel M, Mu DJ, et al. Decreased frontal lobe ratio of N -acetyl aspartate to choline in familial schizophrenia : a proton magnetic resonance spectroscopy study. 2000;289:20–4.

6. Cen H, Xu J, Yang Z, Mei L, Chen T, Zhuo K, et al. Neurochemical and brain functional changes in the ventromedial prefrontal cortex of first-episode psychosis patients: A combined functional magnetic resonance imaging—proton magnetic resonance spectroscopy study. Aust New Zeal J Psychiatry [Internet]. 2020 May 20;54(5):519–27. Available from: http://journals.sagepub.com/doi/10.1177/0004867419898520

7. Corcoran M, Hawkins EL, O’Hora D, Whalley HC, Hall J, Lawrie SM, et al. Are working memory and glutamate concentrations involved in early‐life stress and severity of psychosis? Brain Behav [Internet]. 2020 Jun 9;10(6). Available from: https://onlinelibrary.wiley.com/doi/10.1002/brb3.1616

8. Egerton A, Broberg B V., Van Haren N, Merritt K, Barker GJ, Lythgoe DJ, et al. Response to initial antipsychotic treatment in first episode psychosis is related to anterior cingulate glutamate levels: a multicentre 1H-MRS study (OPTiMiSE). Mol Psychiatry [Internet]. 2018 Jun 7 [cited 2018 Jun 19];1. Available from: http://www.nature.com/articles/s41380-018-0082-9

9. Kraguljac N V., Morgan CJ, Reid MA, White DM, Jindal RD, Sivaraman S, et al. A longitudinal magnetic resonance spectroscopy study investigating effects of risperidone in the anterior cingulate cortex and hippocampus in schizophrenia. Schizophr Res [Internet]. 2019 Aug;210:239–44. Available from: https://linkinghub.elsevier.com/retrieve/pii/S0920996418307242

10. Bartha R, Al-Semaan YM, Williamson PC, Drost DJ, Malla AK, Carr TJ, et al. A short echo proton magnetic resonance spectroscopy study of the left mesial-temporal lobe in first-onset schizophrenic patients. Biol Psychiatry [Internet]. 1999 Jun;45(11):1403–11. Available from: http://linkinghub.elsevier.com/retrieve/pii/S0006322399000074

11. Briend F, Nelson EA, Maximo O, Armstrong WP, Kraguljac N V., Lahti AC. Hippocampal glutamate and hippocampus subfield volumes in antipsychotic-naive first episode psychosis subjects and relationships to duration of untreated psychosis. Transl Psychiatry [Internet]. 2020 Dec 12;10(1):137. Available from: http://www.nature.com/articles/s41398-020-0812-z

12. Galińska-Skok B, Małus A, Konarzewska B, Rogowska-Zach A, Milewski R, Tarasów E, et al. Choline Compounds of the Frontal Lobe and Temporal Glutamatergic System in Bipolar and Schizophrenia Proton Magnetic Resonance Spectroscopy Study. Dis Markers [Internet]. 2018 Nov 25;2018:1–7. Available from: https://www.hindawi.com/journals/dm/2018/3654894/

13. Marsman A, Mandl RCW, Klomp DWJ, Bohlken MM, Boer VO, Andreychenko A, et al. GABA and glutamate in schizophrenia: A 7 T (1)H-MRS study. NeuroImage Clin [Internet]. 2014 Jan [cited 2014 Dec 12];6:398–407. Available from: http://www.pubmedcentral.nih.gov/articlerender.fcgi?artid=4218940&tool=pmcentrez&rendertype=abstract

14. Dempster K, Jeon P, MacKinley M, Williamson P, Théberge J, Palaniyappan L. Early treatment response in first episode psychosis: a 7-T magnetic resonance spectroscopic study of glutathione and glutamate. Mol Psychiatry. 2020;

15. Demjaha A, Egerton A, Murray RM, Kapur S, Howes OD, Stone JM, et al. Antipsychotic treatment resistance in schizophrenia associated with elevated glutamate levels but normal dopamine function. Biol Psychiatry [Internet]. 2014 Mar 1 [cited 2014 Oct 16];75(5):e11-3. Available from: http://www.ncbi.nlm.nih.gov/pubmed/23890739

16. Stanley JA, Williamson CP, Drost DJ, Rylett RJ, Carr TJ, Malta A, et al. An In Vivo Proton Magnetic Resonance Spectroscopy Study of Schizophrenia Patients. Schizophr Bull. 1996;22(4):597–609.

17. Wood SJ, Kennedy D, Phillips LJ, Seal ML, Yücel M, Nelson B, et al. Hippocampal pathology in individuals at ultra-high risk for psychosis: a multi-modal magnetic resonance study. Neuroimage [Internet]. 2010 Aug 1 [cited 2015 Apr 23];52(1):62–8. Available from: http://www.ncbi.nlm.nih.gov/pubmed/20399273

18. Reid M a, Kraguljac N V, Avsar KB, White DM, den Hollander J a, Lahti AC. Proton magnetic resonance spectroscopy of the substantia nigra in schizophrenia. Schizophr Res [Internet]. 2013 Jul [cited 2014 Nov 10];147(2–3):348–54. Available from: http://www.pubmedcentral.nih.gov/articlerender.fcgi?artid=3760722&tool=pmcentrez&rendertype=abstract

19. Wijtenburg SA, Wright SN, Korenic SA, Gaston FE, Ndubuizu N, Chiappelli J, et al. Altered Glutamate and Regional Cerebral Blood Flow Levels in Schizophrenia: A 1H-MRS and pCASL study. Neuropsychopharmacology [Internet]. 2017 Jan 26 [cited 2017 Aug 3];42(2):562–71. Available from: http://www.ncbi.nlm.nih.gov/pubmed/27562377

20. Coughlin JM, Tanaka T, Marsman A, Wang H, Bonekamp S, Kim PK, et al. Decoupling of N-acetyl-aspartate and glutamate within the dorsolateral prefrontal cortex in schizophrenia. Curr Mol Med [Internet]. 2015 [cited 2019 May 21];15(2):176–83. Available from: http://www.ncbi.nlm.nih.gov/pubmed/25732147

21. Bryll A, Krzyściak W, Karcz P, Śmierciak N, Kozicz T, Skrzypek J, et al. The relationship between the level of anterior cingulate cortex metabolites, brain-periphery redox imbalance, and the clinical state of patients with schizophrenia and personality disorders. Biomolecules [Internet]. 2020 Sep 1 [cited 2020 Dec 10];10(9):1–33. Available from: https://pubmed.ncbi.nlm.nih.gov/32899276/

22. Jauhar S, McCutcheon R, Borgan F, Veronese M, Nour M, Pepper F, et al. The relationship between cortical glutamate and striatal dopamine in first-episode psychosis: a cross-sectional multimodal PET and magnetic resonance spectroscopy imaging study. The Lancet Psychiatry. 2018 Oct 1;5(10):816–23.

23. Shirayama Y, Obata T, Matsuzawa D, Nonaka H, Kanazawa Y, Yoshitome E, et al. Specific metabolites in the medial prefrontal cortex are associated with the neurocognitive deficits in schizophrenia: a preliminary study. Neuroimage [Internet]. 2010 Feb 1 [cited 2014 Nov 10];49(3):2783–90. Available from: http://www.ncbi.nlm.nih.gov/pubmed/19850131

24. Theberge J, Bartha R, Drost DJ, Menon RS, Malla A, Takhar J, et al. Glutamate and glutamine measured with 4.0 T proton MRS in never-treated patients with schizophrenia and healthy volunteers. Am J Psychiatry. 2002;159:1944–6.

25. Tibbo PG, Bernier D, Hanstock CC, Seres P, Lakusta B, Purdon SE. 3-T proton magnetic spectroscopy in unmedicated first episode psychosis: a focus on creatine. Magn Reson Med [Internet]. 2013 Mar 1 [cited 2014 Nov 10];69(3):613–20. Available from: http://www.ncbi.nlm.nih.gov/pubmed/22511463

26. Posporelis S, Coughlin JM, Marsman A, Pradhan S, Tanaka T, Wang H, et al. Decoupling of Brain Temperature and Glutamate in Recent Onset of Schizophrenia: A 7T Proton Magnetic Resonance Spectroscopy Study. Biol Psychiatry Cogn Neurosci Neuroimaging [Internet]. 2018 Mar;3(3):248–54. Available from: https://linkinghub.elsevier.com/retrieve/pii/S2451902217300873

27. Goto N, Yoshimura R, Kakeda S, Nishimura J, Moriya J, Hayashi K, et al. Six-month treatment with atypical antipsychotic drugs decreased frontal-lobe levels of glutamate plus glutamine in early-stage first-episode schizophrenia. Neuropsychiatr Dis Treat. 2012;8:119–22.

28. Terpstra M, Vaughan TJ, Ugurbil K, Lim KO, Schulz SC, Gruetter R. Validation of glutathione quantitation from STEAM spectra against edited 1H NMR spectroscopy at 4T: application to schizophrenia. MAGMA [Internet]. 2005 Nov [cited 2015 Apr 27];18(5):276–82. Available from: http://www.ncbi.nlm.nih.gov/pubmed/16320094

29. Rowland LM, Pradhan S, Korenic S, Wijtenburg SA, Hong LE, Edden RA, et al. Elevated brain lactate in schizophrenia: a 7 T magnetic resonance spectroscopy study. Transl Psychiatry [Internet]. 2016 Nov 29 [cited 2017 Feb 21];6(11):e967. Available from: http://www.ncbi.nlm.nih.gov/pubmed/27898072

30. Jessen F, Fingerhut N, Sprinkart AM, Kühn K-U, Petrovsky N, Maier W, et al. N-Acetylaspartylglutamate (NAAG) and N-Acetylaspartate (NAA) in Patients With Schizophrenia. Schizophr Bull [Internet]. 2013 Jan;39(1):197–205. Available from: https://academic.oup.com/schizophreniabulletin/article-lookup/doi/10.1093/schbul/sbr127

31. Yang Z, Zhu Y, Song Z, Mei L, Zhang J, Chen T, et al. Comparison of the density of gamma-aminobutyric acid in the ventromedial prefrontal cortex of patients with first-episode psychosis and healthy controls. Shanghai Arch psychiatry [Internet]. 2015 Dec 25 [cited 2017 Apr 27];27(6):341–7. Available from: http://www.ncbi.nlm.nih.gov/pubmed/27199525

32. Wijtenburg SA, Wang M, Korenic SA, Chen S, Barker PB, Rowland LM. Metabolite Alterations in Adults With Schizophrenia, First Degree Relatives, and Healthy Controls: A Multi-Region 7T MRS Study. Front Psychiatry [Internet]. 2021 May 19;12. Available from: https://www.frontiersin.org/articles/10.3389/fpsyt.2021.656459/full

33. Thomas M, Ke Y, Levitt J, Caplan R, Curran J, Asarnow R, et al. Preliminary study of frontal lobe 1H MR spectroscopy in childhood-onset schizophrenia. J Magn Reson Imaging [Internet]. 1998 [cited 2014 Nov 10];8(4):841–6. Available from: http://onlinelibrary.wiley.com/doi/10.1002/jmri.1880080413/full

34. Smesny S, Berberich D, Gussew A, Schönfeld N, Langbein K, Walther M, et al. Alterations of neurometabolism in the dorsolateral prefrontal cortex and thalamus in transition to psychosis patients change under treatment as usual – A two years follow-up 1H/31P-MR-spectroscopy study. Schizophr Res [Internet]. 2021 Feb;228:7–18. Available from: https://linkinghub.elsevier.com/retrieve/pii/S0920996420306551

35. Sivaraman S, Kraguljac N V., White DM, Morgan CJ, Gonzales SS, Lahti AC. Neurometabolic abnormalities in the associative striatum in antipsychotic-naïve first episode psychosis patients. Psychiatry Res Neuroimaging [Internet]. 2018 Nov;281:101–6. Available from: https://linkinghub.elsevier.com/retrieve/pii/S0925492717303608

36. Kegeles LS, Mao XL, Stanford AD, Girgis R, Ojeil N, Xu XY, et al. Elevated prefrontal cortex γ-aminobutyric acid and glutamate-glutamine levels in schizophrenia measured in vivo with proton magnetic resonance spectroscopy. Arch Gen Psychiatry [Internet]. 2012 May [cited 2014 Sep 30];69(5):449–59. Available from: http://www.ncbi.nlm.nih.gov/pubmed/22213769

37. Coughlin JM, Yang K, Marsman A, Pradhan S, Wang M, Ward RE, et al. A multimodal approach to studying the relationship between peripheral glutathione, brain glutamate, and cognition in health and in schizophrenia. Mol Psychiatry [Internet]. 2021 Jul 19;26(7):3502–11. Available from: https://www.nature.com/articles/s41380-020-00901-5

38. Smesny S, Gussew A, Biesel NJ, Schack S, Walther M, Rzanny R, et al. Glutamatergic dysfunction linked to energy and membrane lipid metabolism in frontal and anterior cingulate cortices of never treated first-episode schizophrenia patients. Schizophr Res [Internet]. 2015 Aug 6 [cited 2015 Aug 11]; Available from: http://www.ncbi.nlm.nih.gov/pubmed/26255566

39. Lutkenhoff ES, van Erp TG, Thomas M a, Therman S, Manninen M, Huttunen MO, et al. Proton MRS in twin pairs discordant for schizophrenia. Mol Psychiatry [Internet]. 2010 Mar [cited 2014 Nov 10];15(3):308–18. Available from: http://www.ncbi.nlm.nih.gov/pubmed/18645571

40. Bustillo JR, Upston J, Mayer EG, Jones T, Maudsley AA, Gasparovic C, et al. Glutamatergic hypo-function in the left superior and middle temporal gyri in early schizophrenia: a data-driven three-dimensional proton spectroscopic imaging study. Neuropsychopharmacology [Internet]. 2020 Oct 13;45(11):1851–9. Available from: http://www.nature.com/articles/s41386-020-0707-y

41. Balz J, Roa Romero Y, Keil J, Schubert F, Ittermann B, Mekle R, et al. Glutamate Concentration in the Superior Temporal Sulcus Relates to Neuroticism in Schizophrenia. Front Psychol [Internet]. 2018 May 7;9. Available from: http://journal.frontiersin.org/article/10.3389/fpsyg.2018.00578/full

42. Liemburg E, Sibeijn-Kuiper A, Bais L, Pijnenborg G, Knegtering H, van der Velde J, et al. Prefrontal NAA and Glx Levels in Different Stages of Psychotic Disorders: a 3T 1H-MRS Study. Sci Rep [Internet]. 2016 Feb 23 [cited 2017 Aug 3];6(1):21873. Available from: http://www.nature.com/articles/srep21873

43. Bartha R, Williamson PC, Drost DJ, Malla A, Carr TJ, Cortese L, et al. Measurement of Glutamate and Glutamine in the Medial Prefrontal Cortex of Never-Treated Schizophrenic Patients and Healthy Controls by Proton Magnetic Resonance Spectroscopy. Arch Gen Psychiatry. 1997;4(10):959–65.

44. Tunc-Skarka N, Weber-Fahr W, Hoerst M, Meyer-Lindenberg A, Zink M, Ende G. MR spectroscopic evaluation of N-acetylaspartate’s T2 relaxation time and concentration corroborates white matter abnormalities in schizophrenia. Neuroimage [Internet]. 2009 Nov 15 [cited 2015 Mar 9];48(3):525–31. Available from: http://www.ncbi.nlm.nih.gov/pubmed/19573608

45. Wood SJ, Berger GE, Wellard RM, Proffitt T, McConchie M, Velakoulis D, et al. A 1H-MRS investigation of the medial temporal lobe in antipsychotic-naïve and early-treated first episode psychosis. Schizophr Res [Internet]. 2008 Jul [cited 2014 Nov 10];102(1–3):163–70. Available from: http://www.ncbi.nlm.nih.gov/pubmed/18456460

46. Onwordi EC, Whitehurst T, Mansur A, Statton B, Berry A, Quinlan M, et al. The relationship between synaptic density marker SV2A, glutamate and N-acetyl aspartate levels in healthy volunteers and schizophrenia: a multimodal PET and magnetic resonance spectroscopy brain imaging study. Transl Psychiatry [Internet]. 2021 Dec 17;11(1):393. Available from: http://www.nature.com/articles/s41398-021-01515-3

47. Stanley J a, Vemulapalli M, Nutche J, Montrose DM, Sweeney J a, Pettegrew JW, et al. Reduced N-acetyl-aspartate levels in schizophrenia patients with a younger onset age: a single-voxel 1H spectroscopy study. Schizophr Res [Internet]. 2007 Jul [cited 2014 Nov 10];93(1–3):23–32. Available from: http://www.pubmedcentral.nih.gov/articlerender.fcgi?artid=2921910&tool=pmcentrez&rendertype=abstract

48. Reid MA, Salibi N, White DM, Gawne TJ, Denney TS, Lahti AC. 7T Proton Magnetic Resonance Spectroscopy of the Anterior Cingulate Cortex in First-Episode Schizophrenia. Schizophr Bull [Internet]. 2018 Jan 29 [cited 2018 Feb 14]; Available from: http://www.ncbi.nlm.nih.gov/pubmed/29385594

49. Choe B, Suh T, Shinn K, Lee C, Paik I. Observation of Metabolic Changes in Chronic Schizophrenia After Neuroleptic Treatment by in vivo hydrogen magnetic resonance spectroscopy. Invest Radiol [Internet]. 1996 [cited 2014 Nov 10];31(6):345–52. Available from: http://scholar.google.com/scholar?hl=en&btnG=Search&q=intitle:Observation+of+Metabolic+Changes+in+Chronic+Schizophrenia+After+Neuroleptic+Treatment#1

50. Taylor R, Osuch EA, Schaefer B, Rajakumar N, Neufeld RWJ, Théberge J, et al. Neurometabolic abnormalities in schizophrenia and depression observed with magnetic resonance spectroscopy at 7 T. BJPsych open [Internet]. 2017 Jan 2 [cited 2017 Mar 1];3(1):6–11. Available from: http://bjpo.rcpsych.org/lookup/doi/10.1192/bjpo.bp.116.003756

51. Batalla A, Bargalló N, Gassó P, Molina O, Pareto D, Mas S, et al. Apoptotic markers in cultured fibroblasts correlate with brain metabolites and regional brain volume in antipsychotic-naive first-episode schizophrenia and healthy controls. Transl Psychiatry [Internet]. 2015 Aug 25;5(8):e626–e626. Available from: http://www.nature.com/articles/tp2015122

52. Chiu PW, Lui SSY, Hung KSY, Chan RCK, Chan Q, Sham PC, et al. In vivo gamma-aminobutyric acid and glutamate levels in people with first-episode schizophrenia: A proton magnetic resonance spectroscopy study. Schizophr Res [Internet]. 2017 Jul 24 [cited 2017 Aug 3]; Available from: http://www.ncbi.nlm.nih.gov/pubmed/28751130

53. Chen T, Wang Y, Zhang J, Wang Z, Xu J, Li Y, et al. Abnormal Concentration of GABA and Glutamate in The Prefrontal Cortex in Schizophrenia.-An in Vivo 1H-MRS Study. Shanghai Arch psychiatry [Internet]. 2017 Oct 25 [cited 2018 Feb 14];29(5):277–86. Available from: http://www.ncbi.nlm.nih.gov/pubmed/29276351

54. Szulc A, Galinska B, Tarasow E, Waszkiewicz N, Konarzewska B, Poplawska R, et al. Proton magnetic resonance spectroscopy study of brain metabolite changes after antipsychotic treatment. Pharmacopsychiatry [Internet]. 2011 Jun [cited 2015 Jan 9];44(4):148–57. Available from: http://www.ncbi.nlm.nih.gov/pubmed/21710405

55. Shakory S, Watts JJ, Hafizi S, Da Silva T, Khan S, Kiang M, et al. Hippocampal glutamate metabolites and glial activation in clinical high risk and first episode psychosis. Neuropsychopharmacology [Internet]. 2018 Oct 28 [cited 2019 Mar 13];43(11):2249–55. Available from: http://www.ncbi.nlm.nih.gov/pubmed/30087434

56. Nenadic I, Maitra R, Basu S, Dietzek M, Schönfeld N, Lorenz C, et al. Associations of hippocampal metabolism and regional brain grey matter in neuroleptic-naïve ultra-high-risk subjects and first-episode schizophrenia. Eur Neuropsychopharmacol [Internet]. 2015 Oct [cited 2017 Aug 10];25(10):1661–8. Available from: http://linkinghub.elsevier.com/retrieve/pii/S0924977X15001546

57. Goldstein ME, Anderson VM, Pillai A, Kydd RR, Russell BR. Glutamatergic Neurometabolites in Clozapine-Responsive and -Resistant Schizophrenia. Int J Neuropsychopharmacol [Internet]. 2015 Apr 6 [cited 2017 May 25];18(6):pyu117–pyu117. Available from: http://www.ncbi.nlm.nih.gov/pubmed/25603859

58. Kim S-Y, Kaufman MJ, Cohen BM, Jensen JE, Coyle JT, Du F, et al. In Viv o Brain Glycine and Glutamate Concentrations in Patients with First-Episode Psychosis Measured by Echo-Time-Averaged Proton MR Spectroscopy at 4 Tesla. Biol Psychiatry [Internet]. 2017 Sep [cited 2017 Sep 19]; Available from: http://linkinghub.elsevier.com/retrieve/pii/S0006322317319418

59. Godlewska BR, Minichino A, Emir U, Angelescu I, Lennox B, Micunovic M, et al. Brain glutamate concentration in men with early psychosis: a magnetic resonance spectroscopy case–control study at 7 T. Transl Psychiatry [Internet]. 2021 Jun 17;11(1):367. Available from: http://www.nature.com/articles/s41398-021-01477-6

60. Gallinat J, McMahon K, Kühn S, Schubert F, Schaefer M. Cross-sectional Study of Glutamate in the Anterior Cingulate and Hippocampus in Schizophrenia. Schizophr Bull [Internet]. 2016 Mar [cited 2017 Aug 9];42(2):425–33. Available from: https://academic.oup.com/schizophreniabulletin/article-lookup/doi/10.1093/schbul/sbv124

61. Kumar J, Liddle EB, Fernandes CC, Palaniyappan L, Hall EL, Robson SE, et al. Glutathione and glutamate in schizophrenia: a 7T MRS study. Mol Psychiatry [Internet]. 2020 Apr 22;25(4):873–82. Available from: http://www.nature.com/articles/s41380-018-0104-7

62. Birur B, Kraguljac NV, VerHoef L, Morgan CJ, Jindal RD, Reid MA, et al. Neurometabolic correlates of 6 and 16 weeks of treatment with risperidone in medication-naive first-episode psychosis patients. Transl Psychiatry [Internet]. 2020 Dec 21;10(1):15. Available from: http://www.nature.com/articles/s41398-020-0700-6

63. Plitman E, Chavez S, Nakajima S, Iwata Y, Chung JK, Caravaggio F, et al. Striatal neurometabolite levels in patients with schizophrenia undergoing long-term antipsychotic treatment: A proton magnetic resonance spectroscopy and reliability study. Psychiatry Res Neuroimaging [Internet]. 2018 Mar 31 [cited 2018 Feb 14];273:16–24. Available from: http://www.ncbi.nlm.nih.gov/pubmed/29414127

64. Bustillo JR, Mayer EG, Upston J, Jones T, Garcia C, Sheriff S, et al. Increased Glutamate Plus Glutamine in the Right Middle Cingulate in Early Schizophrenia but Not in Bipolar Psychosis: A Whole Brain 1H-MRS Study. Front Psychiatry [Internet]. 2021 Jun 7;12. Available from: https://www.frontiersin.org/articles/10.3389/fpsyt.2021.660850/full

65. Borgan FR, Jauhar S, McCutcheon RA, Pepper FS, Rogdaki M, Lythgoe DJ, et al. Glutamate levels in the anterior cingulate cortex in un-medicated first episode psychosis: a proton magnetic resonance spectroscopy study. Sci Rep [Internet]. 2019 Dec 2 [cited 2019 Jul 2];9(1):8685. Available from: http://www.nature.com/articles/s41598-019-45018-0

66. Kraguljac N V, Reid MA, White DM, den Hollander J, Lahti AC. Regional Decoupling of N-acetyl-aspartate and Glutamate in Schizophrenia. Neuropsychopharmacology. 2012;37:2635–42.

67. Ohrmann P, Kugel H, Bauer J, Siegmund A, Kölkebeck K, Suslow T, et al. Learning potential on the WCST in schizophrenia is related to the neuronal integrity of the anterior cingulate cortex as measured by proton magnetic resonance spectroscopy. Schizophr Res [Internet]. 2008 Dec [cited 2014 Nov 10];106(2–3):156–63. Available from: http://www.ncbi.nlm.nih.gov/pubmed/18799290

68. Ota M, Ishikawa M, Sato N, Hori H, Sasayama D, Hattori K, et al. Glutamatergic changes in the cerebral white matter associated with schizophrenic exacerbation. Acta Psychiatr Scand. 2012;126:72–8.

69. Legind CS, Broberg BV, Mandl RCW, Brouwer R, Anhøj SJ, Hilker R, et al. Heritability of cerebral glutamate levels and their association with schizophrenia spectrum disorders: a 1[H]-spectroscopy twin study. Neuropsychopharmacology [Internet]. 2019 Feb 9;44(3):581–9. Available from: http://www.nature.com/articles/s41386-018-0236-0

70. Wood SJ, Yücel M, Wellard RM, Harrison BJ, Clarke K, Fornito A, et al. Evidence for neuronal dysfunction in the anterior cingulate of patients with schizophrenia: a proton magnetic resonance spectroscopy study at 3 T. Schizophr Res [Internet]. 2007 Aug [cited 2014 Nov 10];94(1–3):328–31. Available from: http://www.ncbi.nlm.nih.gov/pubmed/17574388

71. Falkenberg LE, Westerhausen R, Craven AR, Johnsen E, Kroken RA, L⊘berg E-M, et al. Impact of glutamate levels on neuronal response and cognitive abilities in schizophrenia. NeuroImage Clin [Internet]. 2014;4:576–84. Available from: https://linkinghub.elsevier.com/retrieve/pii/S2213158214000448

72. Bartolomeo LA, Wright AM, Ma RE, Hummer TA, Francis MM, Visco AC, et al. Relationship of auditory electrophysiological responses to magnetic resonance spectroscopy metabolites in Early Phase Psychosis. Int J Psychophysiol [Internet]. 2019 Nov;145:15–22. Available from: https://linkinghub.elsevier.com/retrieve/pii/S0167876018311395

73. Hjelmervik H, Craven AR, Sinceviciute I, Johnsen E, Kompus K, Bless JJ, et al. Intra-Regional Glu-GABA vs Inter-Regional Glu-Glu Imbalance: A 1H-MRS Study of the Neurochemistry of Auditory Verbal Hallucinations in Schizophrenia. Schizophr Bull [Internet]. 2020 Apr 10;46(3):633–42. Available from: https://academic.oup.com/schizophreniabulletin/article/46/3/633/5598435

74. Ragland JD, Maddock RJ, Hurtado MY, Tanase C, Lesh TA, Niendam TA, et al. Disrupted GABAergic facilitation of working memory performance in people with schizophrenia. NeuroImage Clin [Internet]. 2020;25:102127. Available from: https://linkinghub.elsevier.com/retrieve/pii/S2213158219304747

75. Ohrmann P, Siegmund A, Suslow T, Spitzberg K, Kersting A, Arolt V, et al. Evidence for glutamatergic neuronal dysfunction in the prefrontal cortex in chronic but not in first-episode patients with schizophrenia: a proton magnetic resonance spectroscopy study. Schizophr Res [Internet]. 2005 Mar 1 [cited 2014 Nov 10];73(2–3):153–7. Available from: http://www.ncbi.nlm.nih.gov/pubmed/15653258

76. Iwata Y, Nakajima S, Plitman E, Caravaggio F, Kim J, Shah P, et al. Glutamatergic Neurometabolite Levels in Patients With Ultra-Treatment-Resistant Schizophrenia: A Cross-Sectional 3T Proton Magnetic Resonance Spectroscopy Study. Biol Psychiatry [Internet]. 2019 Sep 26 [cited 2018 Nov 26];85(7):596–605. Available from: http://www.ncbi.nlm.nih.gov/pubmed/30389132

77. Brandt AS, Unschuld PG, Pradhan S, Lim IAL, Churchill G, Harris AD, et al. Age-related changes in anterior cingulate cortex glutamate in schizophrenia: A (1)H MRS Study at 7 Tesla. Schizophr Res [Internet]. 2016 Apr [cited 2017 Aug 3];172(1–3):101–5. Available from: http://linkinghub.elsevier.com/retrieve/pii/S0920996416300731

78. Ohrmann P, Siegmund A, Suslow T, Pedersen A, Spitzberg K, Kersting A, et al. Cognitive impairment and in vivo metabolites in first-episode neuroleptic-naive and chronic medicated schizophrenic patients: a proton magnetic resonance spectroscopy study. J Psychiatr Res [Internet]. 2007 Oct [cited 2014 Oct 31];41(8):625–34. Available from: http://www.ncbi.nlm.nih.gov/pubmed/16949099

79. Li J, Ren H, He Y, Li Z, Ma X, Yuan L, et al. Anterior Cingulate Cortex Glutamate Levels Are Related to Response to Initial Antipsychotic Treatment in Drug-Naive First-Episode Schizophrenia Patients. Front Psychiatry [Internet]. 2020 Oct 23;11. Available from: https://www.frontiersin.org/articles/10.3389/fpsyt.2020.553269/full

80. Singh S, Khushu S, Kumar P, Goyal S, Bhatia T, Deshpande SN. Evidence for regional hippocampal damage in patients with schizophrenia. Neuroradiology [Internet]. 2018 Feb 11 [cited 2018 Feb 14];60(2):199–205. Available from: http://www.ncbi.nlm.nih.gov/pubmed/29230507

81. Stan a D, Ghose S, Zhao C, Hulsey K, Mihalakos P, Yanagi M, et al. Magnetic resonance spectroscopy and tissue protein concentrations together suggest lower glutamate signaling in dentate gyrus in schizophrenia. Mol Psychiatry [Internet]. 2014 Jun 10 [cited 2014 Nov 5];(August 2013):1–7. Available from: http://www.ncbi.nlm.nih.gov/pubmed/24912493

82. Szulc A, Galińska B, Tarasów E, Walecki J, Dzienis W, Kubas B, et al. [Clinical and neuropsychological correlates of proton magnetic resonance spectroscopy detected metabolites in brains of first-episode and schizophrenic patients]. Psychiatr Pol [Internet]. 2004 Jan [cited 2015 Apr 28];37(6):977–88. Available from: http://www.ncbi.nlm.nih.gov/pubmed/14727370

83. Natsubori T, Inoue H, Abe O, Takano Y, Iwashiro N, Aoki Y, et al. Reduced frontal glutamate + glutamine and N-acetylaspartate levels in patients with chronic schizophrenia but not in those at clinical high risk for psychosis or with first-episode schizophrenia. Schizophr Bull [Internet]. 2014 Sep [cited 2014 Oct 8];40(5):1128–39. Available from: http://www.ncbi.nlm.nih.gov/pubmed/24023251

84. Roalf DR, Nanga RPR, Rupert PE, Hariharan H, Quarmley M, Calkins ME, et al. Glutamate imaging (GluCEST) reveals lower brain GluCEST contrast in patients on the psychosis spectrum. Mol Psychiatry [Internet]. 2017 Sep 24;22(9):1298–305. Available from: http://www.nature.com/articles/mp2016258

85. Kaminski J, Gleich T, Fukuda Y, Katthagen T, Gallinat J, Heinz A, et al. Association of Cortical Glutamate and Working Memory Activation in Patients With Schizophrenia: A Multimodal Proton Magnetic Resonance Spectroscopy and Functional Magnetic Resonance Imaging Study. Biol Psychiatry [Internet]. 2020 Feb;87(3):225–33. Available from: https://linkinghub.elsevier.com/retrieve/pii/S0006322319315446

86. Hutcheson NL, Reid MA, White DM, Kraguljac N V, Avsar KB, Bolding MS, et al. Multimodal analysis of the hippocampus in schizophrenia using proton magnetic resonance spectroscopy and functional magnetic resonance imaging. Schizophr Res. 2012;140:136–42.

87. Kegeles LS, Shungu DC, Anjilvel S, Chan S, Ellis SP, Xanthopoulos E, et al. Hippocampal pathology in schizophrenia: magnetic resonance imaging and spectroscopy studies. Psychiatry Res Neuroimaging [Internet]. 2000 May;98(3):163–75. Available from: http://linkinghub.elsevier.com/retrieve/pii/S0925492700000445

88. Rowland LM, Kontson K, West J, Edden R a, Zhu H, Wijtenburg SA, et al. In vivo measurements of glutamate, GABA, and NAAG in schizophrenia. Schizophr Bull [Internet]. 2013 Sep [cited 2014 Nov 10];39(5):1096–104. Available from: http://www.pubmedcentral.nih.gov/articlerender.fcgi?artid=3756774&tool=pmcentrez&rendertype=abstract

89. Ongür D, Jensen JE, Prescot AP, Stork C, Lundy M, Cohen BM, et al. Abnormal glutamatergic neurotransmission and neuronal-glial interactions in acute mania. Biol Psychiatry [Internet]. 2008 Oct 15 [cited 2015 Jun 26];64(8):718–26. Available from: http://www.pubmedcentral.nih.gov/articlerender.fcgi?artid=2577764&tool=pmcentrez&rendertype=abstract

90. Rowland LM, Summerfelt A, Wijtenburg SA, Du X, Chiappelli JJ, Krishna N, et al. Frontal Glutamate and γ-Aminobutyric Acid Levels and Their Associations With Mismatch Negativity and Digit Sequencing Task Performance in Schizophrenia. JAMA Psychiatry [Internet]. 2016 Feb 1;73(2):166. Available from: http://archpsyc.jamanetwork.com/article.aspx?doi=10.1001/jamapsychiatry.2015.2680

91. Bojesen KB, Broberg BV, Fagerlund B, Jessen K, Thomas MB, Sigvard A, et al. Associations Between Cognitive Function and Levels of Glutamatergic Metabolites and Gamma-Aminobutyric Acid in Antipsychotic-Naïve Patients With Schizophrenia or Psychosis. Biol Psychiatry [Internet]. 2021 Feb;89(3):278–87. Available from: https://linkinghub.elsevier.com/retrieve/pii/S0006322320317376

92. Chang L, Friedman J, Ernst T, Zhong K. Brain metabolite abnormalities in the white matter of elderly schizophrenic subjects: implication for glial dysfunction. Biol … [Internet]. 2007 [cited 2014 Nov 10];62(12):1396–404. Available from: http://www.sciencedirect.com/science/article/pii/S0006322307005215

93. Wang J, Tang Y, Zhang T, Cui H, Xu L, Zeng B, et al. Reduced γ-Aminobutyric Acid and Glutamate+Glutamine Levels in Drug-Naïve Patients with First-Episode Schizophrenia but Not in Those at Ultrahigh Risk. Neural Plast [Internet]. 2016 [cited 2017 Aug 3];2016:3915703. Available from: https://www.hindawi.com/journals/np/2016/3915703/

94. Thakkar KN, Rösler L, Wijnen JP, Boer VO, Klomp DWJ, Cahn W, et al. 7T Proton Magnetic Resonance Spectroscopy of Gamma-Aminobutyric Acid, Glutamate, and Glutamine Reveals Altered Concentrations in Patients With Schizophrenia and Healthy Siblings. Biol Psychiatry [Internet]. 2017 Mar 15 [cited 2017 Apr 27];81(6):525–35. Available from: http://www.ncbi.nlm.nih.gov/pubmed/27316853

95. de la Fuente-Sandoval C, Reyes-Madrigal F, Mao X, León-Ortiz P, Rodríguez-Mayoral O, Jung-Cook H, et al. Prefrontal and Striatal Gamma-Aminobutyric Acid Levels and the Effect of Antipsychotic Treatment in First-Episode Psychosis Patients. Biol Psychiatry [Internet]. 2018 Mar 15 [cited 2018 Feb 14];83(6):475–83. Available from: http://www.ncbi.nlm.nih.gov/pubmed/29132653

96. Bustillo JR, Rowland LM, Mullins P, Jung R, Chen H, Qualls C, et al. 1H-MRS at 4 tesla in minimally treated early schizophrenia. Mol Psychiatry [Internet]. 2010 Jun [cited 2014 Nov 10];15(6):629–36. Available from: http://www.pubmedcentral.nih.gov/articlerender.fcgi?artid=2892215&tool=pmcentrez&rendertype=abstract

97. Rowland LM, Spieker EA, Francis A, Barker PB, Carpenter WT, Buchanan RW. White matter alterations in deficit schizophrenia. Neuropsychopharmacology [Internet]. 2008 [cited 2014 Nov 11];34(6):1514–22. Available from: http://www.nature.com/npp/journal/v34/n6/abs/npp2008207a.html

98. Tayoshi S, Sumitani S, Taniguchi K, Shibuya-Tayoshi S, Numata S, Iga J, et al. Metabolite changes and gender differences in schizophrenia using 3-Tesla proton magnetic resonance spectroscopy (1H-MRS). Schizophr Res [Internet]. 2009 Mar [cited 2014 Nov 10];108(1–3):69–77. Available from: http://www.ncbi.nlm.nih.gov/pubmed/19097753

99. Ongür D, Prescot AP, McCarthy J, Cohen BM, Renshaw PF. Elevated gamma-aminobutyric acid levels in chronic schizophrenia. Biol Psychiatry [Internet]. 2010 Oct 1 [cited 2015 Jun 30];68(7):667–70. Available from: http://www.pubmedcentral.nih.gov/articlerender.fcgi?artid=2942977&tool=pmcentrez&rendertype=abstract

100. Wang AM, Pradhan S, Coughlin JM, Trivedi A, DuBois SL, Crawford JL, et al. Assessing Brain Metabolism With 7-T Proton Magnetic Resonance Spectroscopy in Patients With First-Episode Psychosis. JAMA Psychiatry [Internet]. 2019 Mar 1 [cited 2019 May 15];76(3):314. Available from: http://www.ncbi.nlm.nih.gov/pubmed/30624573

101. Bernier D, Bartha R, McAllindon D, Hanstock CC, Marchand Y, Dillen KNH, et al. Illness versus substance use effects on the frontal white matter in early phase schizophrenia: A 4Tesla 1H-MRS study. Schizophr Res [Internet]. 2016 Aug [cited 2017 Apr 27];175(1–3):4–11. Available from: http://www.ncbi.nlm.nih.gov/pubmed/27161760

102. Xiang Q, Xu J, Wang Y, Chen T, Wang J, Zhuo K, et al. Modular Functional-Metabolic Coupling Alterations of Frontoparietal Network in Schizophrenia Patients. Front Neurosci [Internet]. 2019 Feb 6;13. Available from: https://www.frontiersin.org/article/10.3389/fnins.2019.00040/full

103. Pillinger T, Rogdaki M, McCutcheon RA, Hathway P, Egerton A, Howes OD. Altered glutamatergic response and functional connectivity in treatment resistant schizophrenia: the effect of riluzole and therapeutic implications. Psychopharmacology (Berl) [Internet]. 2019 Jul 28;236(7):1985–97. Available from: http://link.springer.com/10.1007/s00213-019-5188-5

104. Ota M, Wakabayashi C, Sato N, Hori H, Hattori K, Teraishi T, et al. Effect of L-theanine on glutamatergic function in patients with schizophrenia. Acta Neuropsychiatr [Internet]. 2015 Oct 21 [cited 2017 Aug 10];27(5):291–6. Available from: https://www.cambridge.org/core/product/identifier/S0924270815000228/type/journal_article

105. Xin L, Mekle R, Fournier M, Baumann PS, Ferrari C, Alameda L, et al. Genetic Polymorphism Associated Prefrontal Glutathione and Its Coupling With Brain Glutamate and Peripheral Redox Status in Early Psychosis. Schizophr Bull [Internet]. 2016 Sep [cited 2017 Aug 3];42(5):1185–96. Available from: https://academic.oup.com/schizophreniabulletin/article-lookup/doi/10.1093/schbul/sbw038

106. Korenic SA, Klingaman EA, Wickwire EM, Gaston FE, Chen H, Wijtenburg SA, et al. Sleep quality is related to brain glutamate and symptom severity in schizophrenia. J Psychiatr Res [Internet]. 2020 Jan;120:14–20. Available from: https://linkinghub.elsevier.com/retrieve/pii/S0022395619305126

107. Choe B, Kim K, Suh T. 1H magnetic resonance spectroscopy characterization of neuronal dysfunction in drug-naive, chronic schizophrenia. Acad Radiol. 1994;1:211–216.

108. Tarumi R, Tsugawa S, Noda Y, Plitman E, Honda S, Matsushita K, et al. Levels of glutamatergic neurometabolites in patients with severe treatment-resistant schizophrenia: a proton magnetic resonance spectroscopy study. Neuropsychopharmacology [Internet]. 2020 Mar 16;45(4):632–40. Available from: http://www.nature.com/articles/s41386-019-0589-z

109. da Silva Alves F, Boot E, Schmitz N, Nederveen A, Vorstman J, Lavini C, et al. Proton magnetic resonance spectroscopy in 22q11 deletion syndrome. PLoS One [Internet]. 2011 Jan [cited 2014 Nov 10];6(6):e21685. Available from: http://www.pubmedcentral.nih.gov/articlerender.fcgi?artid=3128078&tool=pmcentrez&rendertype=abstract

110. Théberge J, Al-Semaan Y, Williamson PC, Menon RS, Neufeld RWJ, Rajakumar N, et al. Glutamate and glutamine in the anterior cingulate and thalamus of medicated patients with chronic schizophrenia and healthy comparison subjects measured. Am J Psychiatry [Internet]. 2003 [cited 2014 Nov 11];160(12):2231–3. Available from: http://journals.psychiatryonline.org/article.aspx?articleid=176549

111. Galińska B, Szulc A, Tarasów E, Kubas B, Dzienis W, Czernikiewicz A, et al. Duration of untreated psychosis and proton magnetic resonance spectroscopy (1H-MRS) findings in first-episode schizophrenia. Med Sci Monit [Internet]. 2009 [cited 2014 Nov 10];15(2):CR82-R88. Available from: http://www.medscimonit.com/abstract/index/idArt/869559

112. Lesh TA, Maddock RJ, Howell A, Wang H, Tanase C, Daniel Ragland J, et al. Extracellular free water and glutathione in first-episode psychosis—a multimodal investigation of an inflammatory model for psychosis. Mol Psychiatry [Internet]. 2021 Mar 28;26(3):761–71. Available from: http://www.nature.com/articles/s41380-019-0428-y

113. Huang M-L, Khoh T-T, Lu S-J, Pan F, Chen J-K, Hu J-B, et al. Relationships between dorsolateral prefrontal cortex metabolic change and cognitive impairment in first-episode neuroleptic-naive schizophrenia patients. Medicine (Baltimore) [Internet]. 2017 Jun;96(25):e7228. Available from: https://journals.lww.com/00005792-201706230-00048

114. Bryant JE, Lahti AC, Briend F, Kraguljac NV. White Matter Neurometabolic Signatures Support the Deficit and Nondeficit Distinction in Antipsychotic-Naïve First-Episode Psychosis Patients. Schizophr Bull [Internet]. 2021 Jul 8;47(4):1068–76. Available from: https://academic.oup.com/schizophreniabulletin/article/47/4/1068/6161525

115. Chiappelli J, Hong LE, Wijtenburg SA, Du X, Gaston F, Kochunov P, et al. Alterations in frontal white matter neurochemistry and microstructure in schizophrenia: implications for neuroinflammation. Transl Psychiatry [Internet]. 2015 Apr 14;5(4):e548–e548. Available from: http://www.nature.com/articles/tp201543

116. Ćurčić-Blake B, Bais L, Sibeijn-Kuiper A, Pijnenborg HM, Knegtering H, Liemburg E, et al. Glutamate in dorsolateral prefrontal cortex and auditory verbal hallucinations in patients with schizophrenia: A 1 H MRS study. Prog Neuro-Psychopharmacology Biol Psychiatry [Internet]. 2017 Aug;78:132–9. Available from: https://linkinghub.elsevier.com/retrieve/pii/S0278584617300611

117. Jeon P, Limongi R, Ford SD, Mackinley M, Dempster K, Théberge J, et al. Progressive Changes in Glutamate Concentration in Early Stages of Schizophrenia: A Longitudinal 7-Tesla MRS Study. Schizophr Bull Open [Internet]. 2021 Jan 1;2(1). Available from: https://academic.oup.com/schizbullopen/article/doi/10.1093/schizbullopen/sgaa072/6126062

118. Smucny J, Carter CS, Maddock RJ. Magnetic resonance spectroscopic evidence of increased choline in the dorsolateral prefrontal and visual cortices in recent onset schizophrenia. Neurosci Lett [Internet]. 2022 Jan;770:136410. Available from: https://linkinghub.elsevier.com/retrieve/pii/S0304394021007898

119. Huang L-C, Lin S-H, Tseng H-H, Chen KC, Abdullah M, Yang YK. Altered glutamate level and its association with working memory among patients with treatment-resistant schizophrenia (TRS): a proton magnetic resonance spectroscopy study. Psychol Med [Internet]. 2022 Feb 24;1–8. Available from: https://www.cambridge.org/core/product/identifier/S003329172100533X/type/journal_article

120. Leptourgos P, Bansal S, Dutterer J, Culbreth A, Powers A, Suthaharan P, et al. Relating Glutamate, Conditioned, and Clinical Hallucinations via 1H-MR Spectroscopy. Schizophr Bull [Internet]. 2022 Jun 21;48(4):912–20. Available from: https://academic.oup.com/schizophreniabulletin/article/48/4/912/6535483

121. Wang Q, Ren H, Li C, Li Z, Li J, Li H, et al. Metabolite differences in the medial prefrontal cortex in schizophrenia patients with and without persistent auditory verbal hallucinations: a 1H MRS study. Transl Psychiatry [Internet]. 2022 Dec 23;12(1):116. Available from: https://www.nature.com/articles/s41398-022-01866-5

122. Cai X, Pu C, Zhou S, Wang Y, Huang J, Lui SSY, et al. Anterior cingulate glutamate levels associate with functional activation and connectivity during sensory integration in schizophrenia: a multimodal 1 H-MRS and fMRI study. Psychol Med [Internet]. 2022 Jul 6;1–11. Available from: https://www.cambridge.org/core/product/identifier/S0033291722001817/type/journal_article

123. Matrone M, Kotzalidis GD, Romano A, Bozzao A, Cuomo I, Valente F, et al. Treatment-resistant schizophrenia: Addressing white matter integrity, intracortical glutamate levels, clinical and cognitive profiles between early- and adult-onset patients. Prog Neuro-Psychopharmacology Biol Psychiatry [Internet]. 2022 Mar;114:110493. Available from: https://linkinghub.elsevier.com/retrieve/pii/S0278584621002529
